# The lignan niranthin poisons *Leishmania donovani* topoisomerase IB and favours a Th1 immune response in mice

**DOI:** 10.1002/emmm.201201316

**Published:** 2012-10-02

**Authors:** Sayan Chowdhury, Tulika Mukherjee, Rupkatha Mukhopadhyay, Budhaditya Mukherjee, Souvik Sengupta, Sharmila Chattopadhyay, Parasuraman Jaisankar, Syamal Roy, Hemanta K Majumder

**Affiliations:** 1 Molecular Parasitology Laboratory, Indian Institute of Chemical BiologyJadavpur, Kolkata, India; 2 Department of Chemistry, Indian Institute of Chemical BiologyJadavpur, Kolkata, India; 3 Infectious Diseases and Immunology Division, Indian Institute of Chemical BiologyJadavpur, Kolkata, India; 4 Drug Development Diagnostics and Biotechnology Division, Indian Institute of Chemical BiologyJadavpur, Kolkata, India

**Keywords:** antimony resistance, chemotherapy, *Leishmania donovani*, niranthin, topoisomerase

## Abstract

Niranthin, a lignan isolated from the aerial parts of the plant *Phyllanthus amarus*, exhibits a wide spectrum of pharmacological activities. In the present study, we have shown for the first time that niranthin is a potent anti-leishmanial agent. The compound induces topoisomerase I-mediated DNA–protein adduct formation inside *Leishmania* cells and triggers apoptosis by activation of cellular nucleases. We also show that niranthin inhibits the relaxation activity of heterodimeric type IB topoisomerase of *L. donovani* and acts as a non-competitive inhibitor interacting with both subunits of the enzyme. Niranthin interacts with DNA–protein binary complexes and thus stabilizes the ‘cleavable complex’ formation and subsequently inhibits the religation of cleaved strand. The compound inhibits the proliferation of *Leishmania* amastigotes in infected cultured murine macrophages with limited cytotoxicity to the host cells and is effective against antimony-resistant *Leishmania* parasites by modulating upregulated P-glycoprotein on host macrophages. Importantly, besides its *in vitro* efficacy, niranthin treatment leads to a switch from a Th2- to a Th1-type immune response in infected BALB/c mice. The immune response causes production of nitric oxide, which results in almost complete clearance of the liver and splenic parasite burden after intraperitoneal or intramuscular administration of the drug. These findings can be exploited to develop niranthin as a new drug candidate against drug-resistant leishmaniasis.

## INTRODUCTION

Leishmaniasis is one of the most serious forms of parasitic diseases caused by the protozoan flagellates of the genus *Leishmania*. The disease presents a spectrum of clinical manifestations (Olivier et al, 2005) ranging from ulcerative skin lesions developing at the site of the sandfly bite (localized cutaneous leishmaniasis) to the most destructive mucosal inflammation and disseminated visceral infection (Reithinger et al, 2007). More than 350 million people are at risk of the infection and the disease causes about 70,000 deaths each year globally (Kaye & Scott, 2011). To date, there is no vaccine against any form of human leishmaniasis (Nagill & Kaur, 2011). For the last six decades, organic pentavalent antimonials [Sb (V)] have been the first line drugs for the treatment of this disease and emergence of resistant clinical isolates to these drugs pose a serious obstacle for disease control and treatment (Ashutosh & Goyal, 2007). Alternative medicines like amphotericin B (Wortmann et al, 2010) or miltefosine (Rahman et al, 2011) are in phase IV trials despite their costliness and teratogenicity. Thus, the identification of new molecular targets for improved therapy of *Leishmania* infection is vital.

DNA topoisomerases are ubiquitous DNA-manipulating enzymes, which catalyse the breakage and rejoining of DNA strands to modulate the dynamic nature of DNA secondary and higher order structures in various vital life processes involving DNA transactions (Liu, 1989; Wang, 1996). Topoisomerases can be classified into two groups based on their DNA processing abilities: type I and type II, both of which are of equal importance as chemotherapeutic targets (Fortune & Osheroff, 2006; Pommier, 2006). In contrast to other eukaryotic topoisomerases, *L. donovani* topoisomerase I is an unusual heterodimeric enzyme in which the core DNA binding domain ‘VAILCNH’ is located on the large subunit (LdTOP1L, 635 amino acids) and catalytic domain harbouring the catalytic ‘SKXXY’ motif is located on the small subunit (LdTOP1S, 262 amino acids; Das et al, 2004). DNA topoisomerases have emerged as principal therapeutic targets and targeting agents have a broad spectrum of anti-parasitic activity. These inhibitors can be broadly classified into two classes. The class I inhibitors stabilize the formation of topoisomerase–DNA covalent complex (cleavable complex) and are ‘topoisomerase poisons’ while the inhibitors, which abrogate only the catalytic property of the enzyme and thus interfere with the formation of ‘covalent complex’ formation are called ‘class II inhibitors’ (Chowdhury et al, 2011).

The lignan family of natural products includes compounds with important anti-neoplastic and anti-viral properties. Podophyllotoxin and two other semisynthetic derivatives, etoposide and teniposide, are common amongst them, and they show a wide variety of anti-cancer properties (Gordaliza et al, 2000) by inhibiting topoisomerase II and microtubule assembly (Hartmann & Lipp, 2006; Imbert, 1998). The lignan-rich fraction from aerial parts of *Phyllanthus amarus* exhibits cytotoxic effects in the K-562 cell line and contains niranthin, which has the most potent inhibitory activity (Leite et al, 2006). Niranthin also exhibits anti-inflammatory and anti-allodynic properties (Kassuya et al, 2006) and has been shown to possess anti-viral activity against human hepatitis B virus *in vitro* (Huang et al, 2003).

In the present study, we have demonstrated for the first time that the lignan niranthin isolated from *P. amarus* is an anti-leishmanial agent and induces apoptotic events in the parasites. Induction of reactive oxygen species (ROS) formation and activation of nucleases lead to DNA fragmentation and the compound forms a cleavage complex with topoisomerase I of *L. donovani*. Our data suggest that niranthin binds with each subunit of the enzyme. This novel topoisomerase IB poison of *L. donovani* can efficiently reduce parasite burden in cultured macrophages infected with antimony-resistant and -sensitive parasites. Niranthin at low concentration, when combined with sodium antimony gluconate (SAG), can clear SAG-unresponsive *Leishmania* (GE1) by reversing multidrug resistance. In addition, as an immunomodulatory molecule *in vivo*, niranthin directs the immune response from the parasite-protecting Th2 to the host-protective Th1 response. This results in IL-12-driven expansion of Th1 cells and macrophage activation through the production of IFN-γ, which ultimately triggers generation of nitric oxide (NO) and ROS in macrophages and thus confers almost complete protection against leishmaniasis in BALB/c mice. Taken together, niranthin is a promising new therapeutic agent against emerging antimony-resistant strains of *Leishmania*.

## RESULTS

### Niranthin inhibits growth of *L. donovani* promastigotes by induction of apoptosis

**Figure 1:**
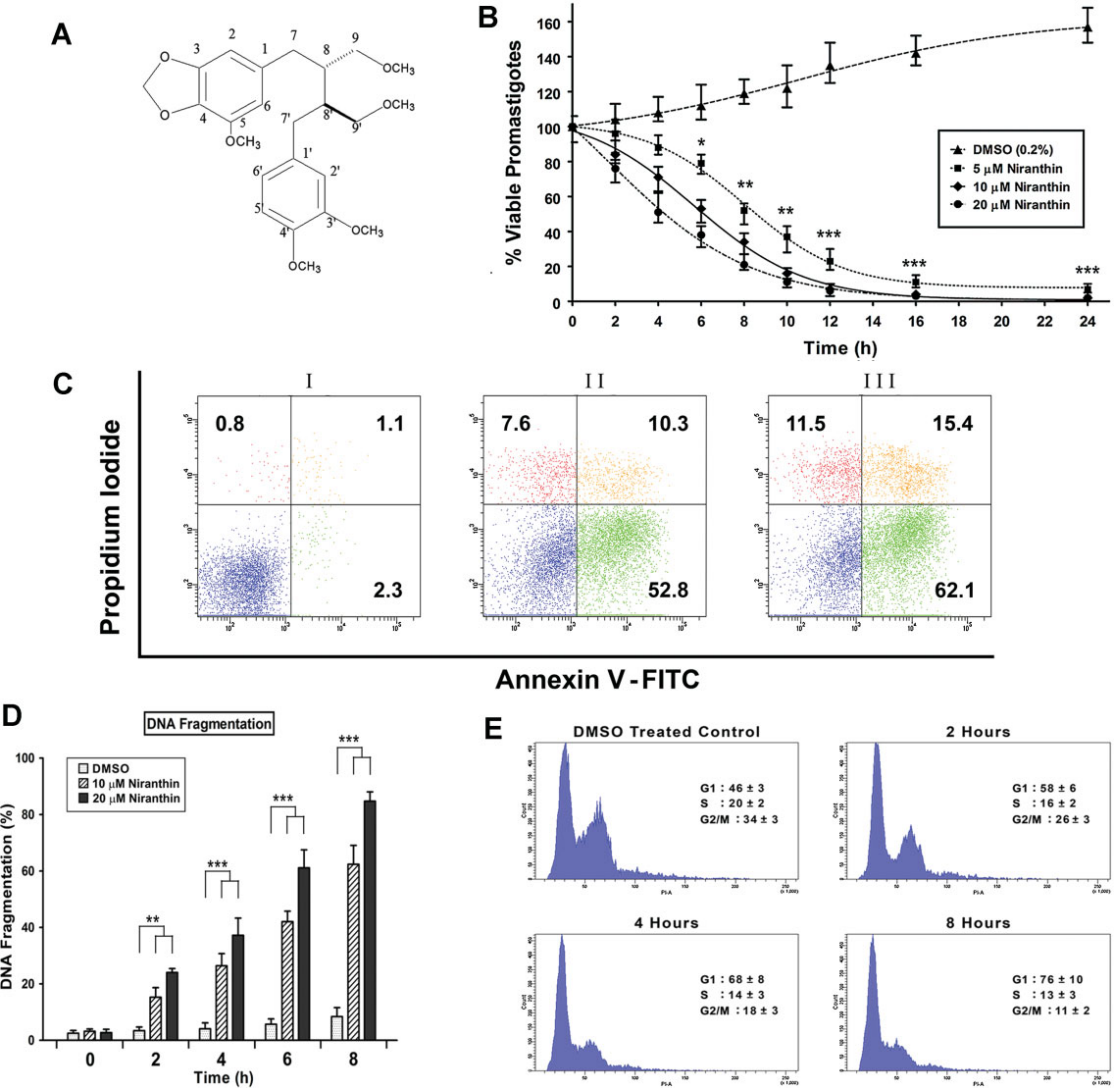
Cytotoxicity analysis and morphological and physiological changes associated with niranthin-mediated cell death Structure of niranthin.Promastigotes were cultured in the presence of 0.2% DMSO (▴) or 5 µM (▪), 10 µM (♦) and 20 µM (•) of niranthin. Aliquots were taken at intervals and percentage of viable promastigotes was measured by Alamar Blue reagent. Values were taken in triplicate, averaged and plotted against time. Data represent means ± SE (*n* = 3). *p < 0.05, **p < 0.01 (Student's *t*-test). ***Indicates significant difference between DMSO control and 5, 10 or 20 µM niranthin treatment (*p* < 0.001) at the 12 h time-point.Flow cytometric analysis of promastigote death through PCD/necrotic processes. Parasites were stained with FITC-annexinV and propidium iodide after treatment with 0.2% DMSO (panel I), niranthin (10 µM; panel II) for 6 and 8 h (panel III), respectively.Extent of genomic DNA fragmentation upon niranthin treatment. Parasites were treated with 10 and 20 µM of niranthin for the indiacted time periods. As negative control, parasites were also treated with 0.2% DMSO. Values were obtained from the MULTISCAN EX readings at 405 nm. The percentage was plotted as units of time. Data represent means ± SD (*n* = 3). **p < 0.01 (Student's *t*-test). ***Indicates significant difference between DMSO control and 20 µM niranthin treatment (*p* < 0.001) for 4, 6 and 8 h.Niranthin-mediated cell cycle arrest in *L. donovani* AG83 promastigote cells. Histograms of distribution of DNA content with flow cytometry in *Leishmania* cells. Cell cycle arrest was analysed after treatment with 0.2% DMSO as control, and with niranthin (20 µM) for 2, 4 and 8 h. Cells were then fixed and stained with propidium iodide, and nuclei were analysed for DNA content by flow cytometry. In total, 20,000 nuclei were counted from each sample. The percentages of cells within different cells stages were determined as described in Materials and Methods.

To evaluate the anti-leishmanial potential of niranthin (Fig 1A), we first investigated whether the compound induces cell death in the parasite. Promastigotes were treated with different concentrations of niranthin and cell viability measured. After 24 h, viability was reduced by 93 and 98% using 5 and 10 µM niranthin, respectively (Fig 1B). In addition, treatment with niranthin generated a fivefold higher amount of ROS compared to DMSO treatment inside the parasites (Supporting Information Fig S1). The mode of cell death in niranthin-treated parasites was investigated by fluorescein isothiocyanate (FITC)-annexinV and propidium iodide (PI) staining. Externalization of phosphatidyl serine (stained by annexinV) and presence of impermeant cell membrane (negative PI staining) are hallmarks of programmed cell death (PCD). Flow cytometry shows that 96% of DMSO-treated parasites were both PI- and annexinV-negative (Fig 1C, panel I), while 53% of niranthin-treated parasites were annexinV-positive after 6 h of drug treatment (10 µM; Fig 1C, panel II). A time-dependent increase of both FITC-annexinV-positive cells and double-positive (FITC-annexinV and PI) populations were observed (Fig 1C, panel III), which indicates that the drug-treated cells die via the apoptotic pathway. The extent of DNA fragmentation in niranthin-treated parasites was estimated using an ELISA and a time-dependent increase in niranthin-induced DNA fragmentation up to 85% at 8 h was observed (Fig 1D). This indicates that the triggering of apoptosis ultimately activates different nucleases to degrade the parasite nucleosomal structure.

With the degradation of oligonucleosomal structures and induction of apoptosis, treatment with niranthin reduces replication of the parasites and eventually slows down the active cell cycle. Untreated cells show the expected cell cycle distribution in G1, S and G2/M phases throughout the experiment (G1: 46%, S: 20% and G2/M: 34%) while treatment with niranthin caused arrest at G1 phase and inhibited entry into S phase of *L. donovani* promastigotes (Fig 1E). Prolonged treatment for 8 h dramatically reduces the G2/M population (11%) compared to control cells. As a result, parasite replication is hampered in a time-dependent manner (G2/M population reduced from 26 to 11%).

### Niranthin stabilizes topoisomerase cleavable complex formation in *L. donovani* promastigotes *in vivo* and *in vitro*

**Figure 2:**
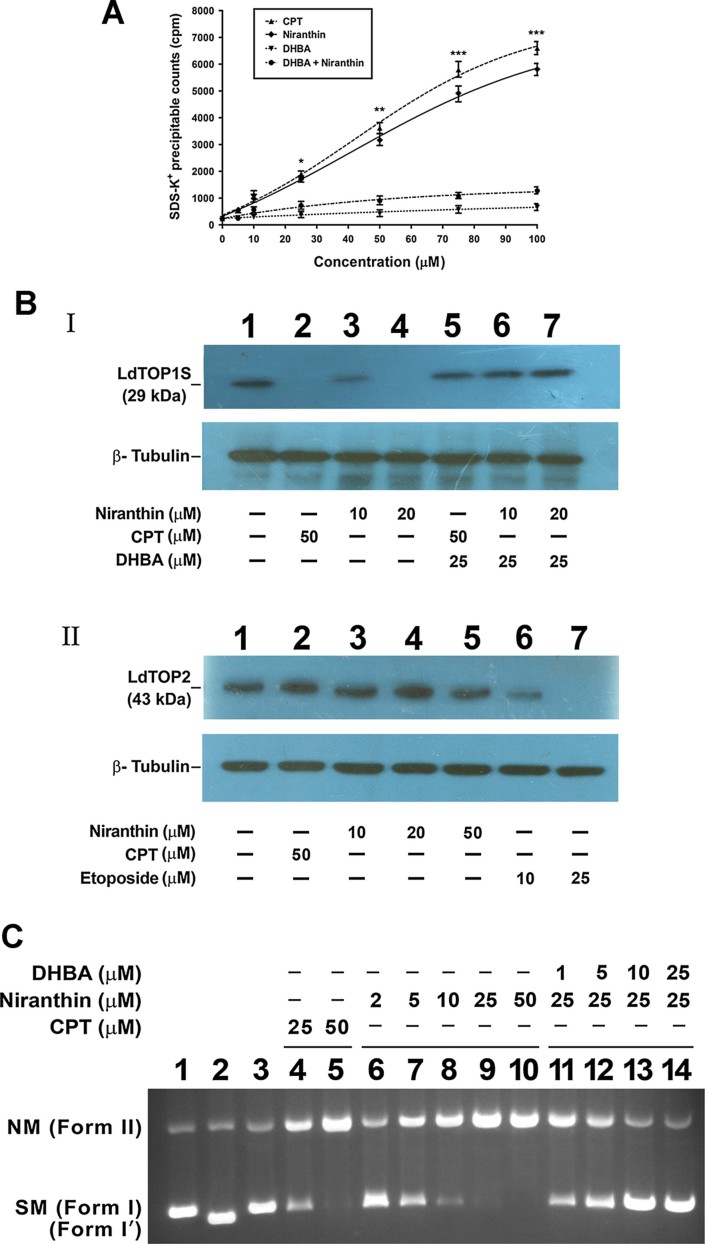
Analysis of protein linked DNA complex formation in *L. donovani* promastigotes Exponentially growing *L. donovani* promastigotes (5 × 10^6^ cells/ml) were labeled with [^3^H] thymidine at 22°C for 24 h and then treated with different concentrations of niranthin and DHBA as indicated. SDS-K^+^ precipitable complex were measured as described in Materials and Methodss. Experiments were performed three times and representative data from one set of experiments are expressed as means ± SE. Variations among different set of experiments were <10%. *p < 0.05, **p < 0.01 (Student's *t*-test). ***Indicates significant difference between DHBA control and niranthin treatments (*p* < 0.001).Stabilization of topoisomerase-mediated cleavable complex was analysed by the immunoband depletion assay. Panel I: immunoband depletion of *L. donovani* topoisomerase I using an antibody raised against LdTOP1S. Leishmanial cells were treated with 0.2% DMSO alone (lane 1); with 10 and 20 µM of niranthin (lanes 3 and 4); 50 µM CPT (lane 2) and 25 µM DHBA before treatment with niranthin (lanes 6 and 7) or CPT (lane 5). Panel II: immunoband depletion of *L. donovani* topoisomerase II using an antibody raised against ATPase domain of *L. donovani* topoisomerase II. The cells were treated with 0.2% DMSO alone (lane 1) or with 10, 20 and 50 µM niranthin (lanes 3–5); 50 µM of CPT (lane 2) and 10 and 25 µM etoposide (lanes 6 and 7). Panel III: β-tubulin served as loading control.Cleavage reaction and agarose gel electrophoresis were performed as described in the Experimental section. Lane 1, 50 fmol of pHOT1 DNA; lane 2, with 100 fmol LdTOP1LS; lane 3, same as lane 2, but with SDS–proteinase K treatment; lanes 4 and 5, same as lane 3, but in the presence of 25 and 50 µM CPT, respectively, as control; lanes 6–10, same as lane 3, but in the presence of 2, 5, 10, 25 and 50 µM niranthin; lanes 11–14, same as lane 2, but the enzyme was pre-incubated with 1, 5, 10 and 25 µM DHBA before addition of niranthin (25 µM) and DNA. Positions of supercoiled monomer (SM; form I) and nicked monomer (NM; form II) are indicated. Form I′, relaxed molecules.

The ability of niranthin to induce protein-linked DNA complexes, which could lead to apoptosis in the *L. donovani* promastigotes, was investigated by KCl-SDS precipitation assay (Bodley & Shapiro, 1995). The experiments were performed with [^3^H]-thymidine-labelled promastigotes, treated with various concentrations of niranthin as well as camptothecin (CPT) and dihydrobetulinic acid (DHBA) as controls. Treatment of cells with different concentrations of niranthin significantly increased the SDS–K^+^-precipitable complex compared with the untreated control cells (Fig 2A) and their amount was similar to that obtained by treatment with different concentrations of CPT for 8 h. These results indicated that the formation of SDS–K^+^ precipitable complexes is due to formation of protein-linked DNA complexes following treatment with niranthin. Pre-treatment for 30 min with 200 µM DHBA, which antagonizes topoisomerase I-mediated DNA cleavage, before incubation with niranthin prevents accumulation of precipitable complexes, which strongly suggests that their accumulation induced by niranthin is due to topoisomerase–DNA links.

To support the notion of covalent complex formation between topoisomerase I and DNA in intact cells, we carried out immunoband depletion experiments with *L. donovani* promastigotes. Nuclear fractions were prepared from untreated as well as drug-treated promastigotes and subjected to SDS–PAGE. If topoisomerase I can form a covalent complex with genomic DNA inside cells, this topoisomerase I–DNA covalent complex cannot penetrate into the gel. On the other hand, if topoisomerase I does not form a complex with DNA and remains free, it will enter into the gel. The immunoband of topoisomerase I gradually disappeared with increasing concentration of niranthin during an 8 h incubation (Fig 2B, panel I, lanes 3 and 4) similar to treatment with 50 µM of CPT for 8 h (lane 2). Pre-incubation with 20 µM of DHBA before treatment with niranthin (Fig 2B, panel I, lanes 6 and 7) prevents immunoband depletion of *L. donovani* topoisomerase I as cleavable complex formation is prevented. The same experiment was performed with an antibody against LdTOP2 to investigate if niranthin could enhance *in vivo* cleavable complex formation by topoisomerase II. The topoisomerase II immunoband was not depleted by increasing the concentration of niranthin (10–50 µM) during the same time period of incubation (Fig 2B, panel II, lanes 3–5) compared to treatment with etoposide (Fig 2B, panel II, lanes 6 and 7), which is a known topoisomerase II poison. The above results suggest that niranthin treatment leads to stabilization of topoisomerase I–DNA cleavable complex formation inside leishmanial cells.

To further investigate the cleavage complex stabilization by niranthin, *in vitro* plasmid cleavage reactions were performed under equilibrium conditions by reacting LdTOP1LS with pHOT1 DNA, which harbours a topoisomerase IB-specific binding site, in the presence of niranthin and CPT (Fig 2C) in presence of increasing concentrations of either CPT or niranthin. We observed that closed circular DNA (form I) was converted to nicked circular DNA (form II) in presence of 100 fmol of LdTOP1LS with increasing drug concentrations (Fig 2C, lanes 6–10). The formation of form II DNA in the presence of topoisomerase I only is shown in lane 3. This result shows that niranthin does not inhibit the cleavage step of the topoisomerase I-catalysed reaction, but stabilizes the topoisomerase I-mediated cleavable complex and acts as a topoisomerase poison. Furthermore, addition of increasing concentrations of DHBA prior to the addition of 25 µM of niranthin (Fig 2C, lanes 11–14) caused inhibition of niranthin-mediated plasmid cleavage. The above observation was also supported by duplex oligonucleotide cleavage assay (Supporting Information Fig S2) where the stabilization of a covalent complex (formed by niranthin) between 25-mer duplex DNA and LdTOP1LS was similar to that of CPT.

Catalytic assays do not allow a precise chronological dissection of the inhibitory mechanism in relation to the catalytic cycle. Thus, the step at which an inhibitor needs to enter the catalytic cycle, and the step at which it becomes effective in trapping or inhibiting the enzyme cannot be differentiated. In order to overcome this problem, we generated an oligonucleotide suicide substrate of topoisomerase IB, which restricts the enzyme to a single round of cleavage and religation and allows for addressing the two half-reactions separately as we have described previously (Das et al, 2006). Like CPT, niranthin inhibits the religation reaction, when added to the enzyme substrate covalent complex that had been previously formed in the absence of the drug (Supporting Information Fig S3, lanes 10 and 11) after the suicidal cleavage reaction. These data suggest that the interaction of niranthin with the enzyme during the trans-esterification reaction with DNA is a pre-requirement for the stabilization of the topoisomerase I-cleavable complex.

### Niranthin inhibits the catalytic activity of *L. donovani* topoisomerase IB and acts like a non-competitive inhibitor

**Figure 3:**
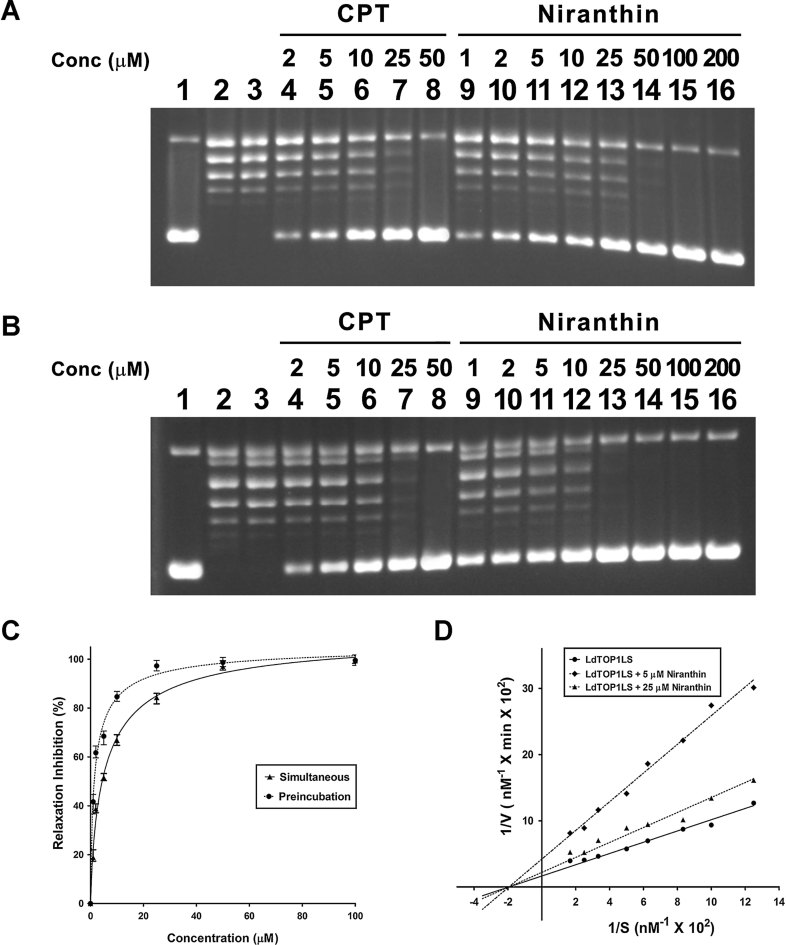
Inhibition of relaxation assay by niranthin Relaxation of supercoiled pBS (SK^+^) DNA with reconstituted LdTOP1LS at a molar ratio of 3:1. Lane 1, 90 fmol of pBS (SK^+^) DNA; lane 2, same as lane 1, but simultaneously incubated with 30 fmol of LdTOP1LS for 30 min at 37°C; lane 3, same as lane 2, but in presence of 2% v/v DMSO; lanes 4–8, same as lane 2, but in presence of 2, 5, 10, 25 and 50 µM of CPT, respectively; lanes 9–16, same as lane 2, but in presence of 1, 2, 5, 10, 25, 50, 100 and 200 µM of niranthin, respectively.Preincubation of LdTOP1LS with respective inhibitors followed by addition of DNA. Lane 1, 90 fmol of pBS (SK^+^) DNA; lanes 2 and 3, same as lane 1, but DNA was added after preincubation of 30 fmol LdTOP1LS with reaction buffer and with 2% DMSO, respectively, for 5 min at 37°C; lanes 4–8, same as lane 2, but the enzyme was pre-incubated with 2, 5, 10, 25 and 50 µM CPT, respectively; lanes 9–16, same as lane 2, but the enzyme was pre-incubated with 1, 2, 5, 10, 25, 50, 100 and 200 µM niranthin, respectively.Quantitative representation of enzyme inhibition as a function of inhibitor concentrations in presence of niranthin in relaxation experiments. The fitted lines (sigmoidal) from these data points (*n* = 3) have the *R*^2^ values of 0.9828 and 0.9793 respectively.Lineweaver–Burk representation of the kinetics of relaxation of negatively supercoiled pBS (SK^+^) DNA by LdTOP1LS alone (•) or with 5 µM (▴) and 25 µM (♦) of niranthin.

The overall effect of niranthin on the unusual bi-subunit type IB topoisomerase of *L. donovani* (LdTOP1LS) was examined by plasmid relaxation assays in which the molar ratio of plasmid DNA and enzyme was 3:1. Under this condition in absence of any inhibitor, LdTOP1LS relaxes supercoiled plasmid DNA completely after 20 min of incubation (Roy et al, 2008). The inhibition of this reaction by niranthin increases in a dose-dependent manner (Fig 3A, lanes 9–16). At 25 µM, almost 85% inhibition was achieved (Fig 3A, lane 13) while 98% inhibition was achieved by 50 µM niranthin (lane 14).

To investigate the interaction of niranthin with the enzyme in this relaxation experiment, LdTOP1LS was pre-incubated with niranthin at different concentrations prior to addition of substrate plasmid DNA (Fig 3B). The inhibition of LdTOP1LS by pre-incubation with niranthin was compared with the inhibitory effects of niranthin incubated simultaneously with the enzyme and supercoiled DNA in the relaxation assay. Under these conditions, 85% inhibition was achieved at 10 µM niranthin (Fig 3B, lane 12) and 98% inhibition at 25 µM (Fig 3B, lane 13).

The percentage of inhibition of relaxation was plotted against the concentration of the compounds for both simultaneous and preincubation assay conditions (Fig 3C). The IC_50_ values of niranthin in simultaneous and pre-incubation DNA relaxation assay were 4.86 and 1.52 µM, respectively, using the variable slope model, which fits the nonlinear regression equation.

Camptothecin, the well established inhibitor of LdTOP1LS, acts as a non-competitive inhibitor of topoisomerase IB (Roy et al, 2008). The above pre-incubation relaxation assay and cleavage experiment suggest that niranthin interacts with the DNA-bound as well as the free enzyme. To further investigate the inhibition of LdTOP1LS by niranthin, a time course relaxation experiment was performed in which the concentration of supercoiled substrate pBS (SK^+^) DNA was varied over a range of 8–60 nM. The velocity of the enzyme remains linear for the first 5 min of reaction and all the subsequent velocities for this kinetic study were measured for up to 1 min, which falls within the linear range for the velocity examined. The initial velocities for each substrate concentration were plotted in a Lineweaver–Burk plot (Fig 3D). The maximal velocity (*V*_max_) for the LdTOP1LS was 6.37 × 10^−8^ M base pairs of supercoiled DNA relaxed/min/0.98 nM of enzyme, which corresponds to a turnover of about 65 plasmid molecules relaxed/min/molecules of enzyme and is reduced upon the incubation of different concentrations of niranthin with LdTOP1LS.

### Niranthin binds the enzyme in reversible manner and interacts with each subunit of LdTOP1LS in equimolar fashion

**Figure 4:**
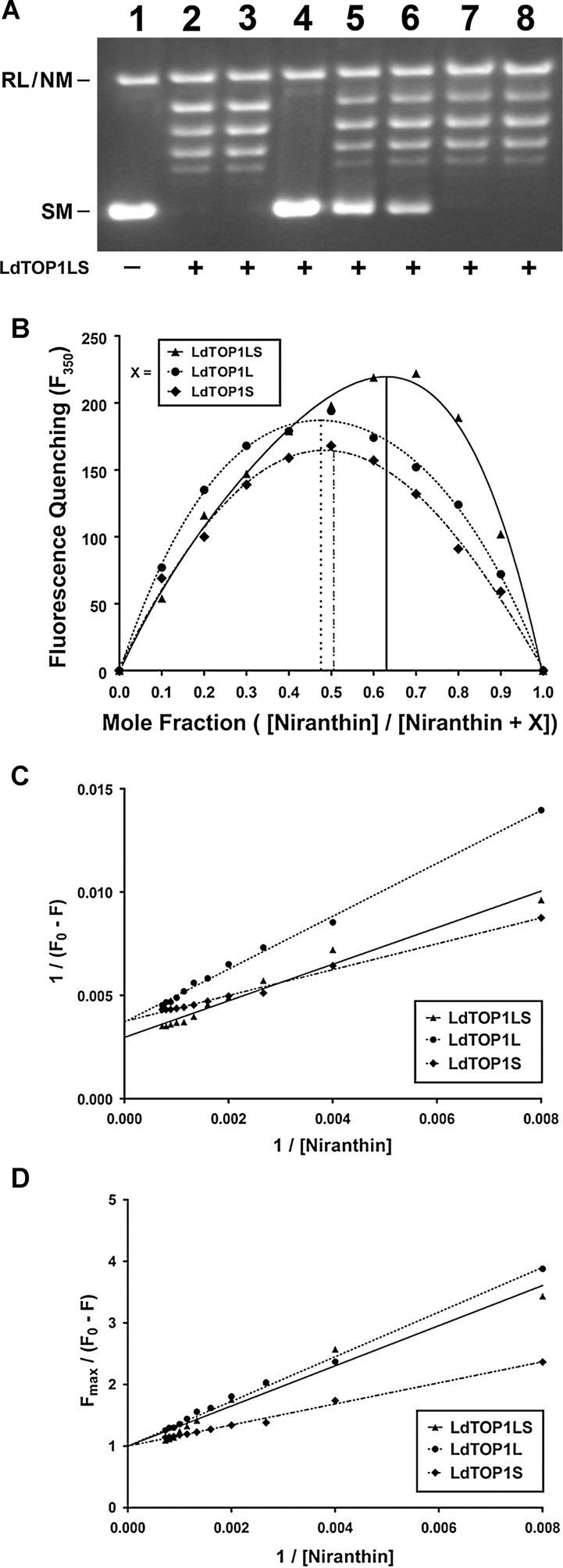
Reversible and weak interaction between nirathin and LdTOP1LS Niranthin binds with LdTOP1LS in reversible manner. Lane 1, 50 fmol of pBS (SK^+^) DNA. Lane 2, LdTOP1LS (100 fmol) incubated at 37°C for 3 min in the reaction mixture before addition of pBS (SK^+^) DNA. Lane 3, same as lane 2, but in presence of 2% v/v DMSO. Lanes 4, same as lane 2, but in presence of 20 µM of niranthin, pre-incubated with LdTOP1LS for 3 min at 37°C in relaxation buffer followed by addition of 50 fmol of pBS (SK^+^) DNA and further incubated for 15 min at 37°C. Lane 5–7, same as lane 4, but diluted to 5-, 10- and 20-fold so that the final inhibitor concentrations became 4, 2 and 1 µM of niranthin. These were followed by addition of pBS (SK^+^) DNA and further incubated for 10 min at 37°C. Lane 8, same as lane 2, but in presence of 1 µM of niranthin before addition of DNA. The experiments were performed three times and representative result is from one set of experiments.Job's Plot of LdTOP1LS (▴), LdTOP1L (•) and LdTOP1S (♦) (as indicated by X) binding to niranthin.Double reciprocal plot of inhibitor binding to LdTOP1LS (▴), LdTOP1L (•) and LdTOP1S (♦).The linear plot of binding of niranthin to LdTOP1LS (▴), LdTOP1L (•) and LdTOP1S (♦).

The inhibition of the relaxation reaction after pre-incubation as described above supports that niranthin interacts with the enzyme. However, it remained unclear whether the interaction is strong enough so that they can act on the enzyme in an irreversible manner. This critical issue was investigated in a dilution experiment. Reconstituted LdTOP1LS was preincubated with 20 µM of niranthin (Fig 4A, lane 4), the concentration at which 95–99% inhibition of enzyme activity has been achieved. The reaction mixtures were subsequently diluted fivefold so that the final concentration was 4 µM niranthin at which partial (about 30%) relief of inhibition was observed (Fig 4A, lane 5). Further 10-fold (Fig 4A, lane 6) and 20-fold (Fig 4A, lane 7) dilution resulted in almost 80% and complete relief of inhibition, respectively. In the drug control reaction, *i.e.* inhibition with 1 µM niranthin showed the expected pattern of inhibition (Fig 4A, lanes 8). Thus, the relief of inhibition upon dilution suggests that niranthin acts in reversible fashion against LdTOP1LS.

To provide insight the nature of the enzyme–inhibitor interaction, the binding of niranthin to LdTOP1LS was investigated by measuring the quenching of intrinsic tryptophan fluorescence of each of the proteins individually. The stoichiometry of the ligand–protein interaction was measured using Job plot (Huang, 1982). The concentration of both LdTOP1LS and niranthin was continuously varied keeping the total ligand–protein concentration fixed at 1.25 µM. The excitation and emission slit widths were 5 and 10 nm, respectively. The stoichiometry of binding was found to be 2:1 for LdTOP1LS, which suggests that there are two binding sites for niranthin in the enzyme (Fig 4B). Further studies with each subunit of LdTOP1LS (*i.e.* LdTOP1L and LdTOP1S) confirms that niranthin binds separately to each of the subunits in equimolar concentration (1:1; Fig 4B).

The dissociation constant was calculated from Fig 4C and D. Figure 4C shows the quenching profile of a fixed amount of LdTOP1LS, LdTOP1L and LdTOP1S (200 nM) with varying concentrations of niranthin (0–15 µM). The dissociation constant (*K*_D_) of the ligand–enzyme interaction was determined and is given in Table 1.

**Table 1:** Dissociation constant of niranthin with LdTOP1LS, LdTOP1L and LdTOP1S

Protein	*K*_D_ (µM)
LdTOP1LS	0.299 ± 0.16
LdTOP1L	0.372 ± 0.26
LdTOP1S	0.169 ± 0.12

### Niranthin inhibits growth of *L. donovani* intracellular amastigotes and reverses multidrug resistance (MDR1) in macrophages infected with sodium antimony gluconate (SAG)-resistant parasites

**Figure 5:**
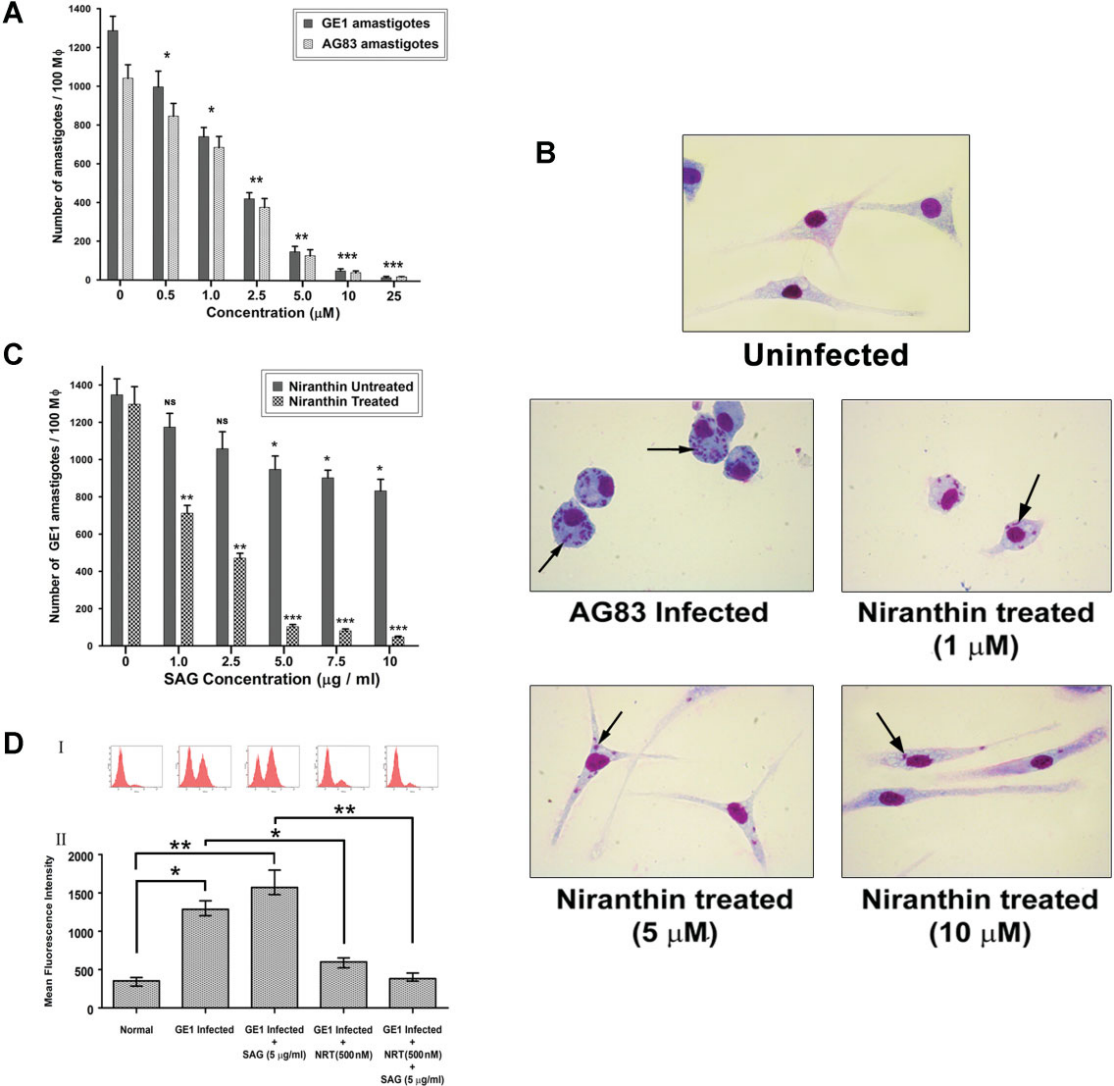
Clearance of Sb^S^ *L. donovani* (AG83) and Sb^R^ *L. donovani* (GE1) parasites from infected mouse macrophages by reversal of multidrug resistance Dose dependent clearance of AG83 amastigotes (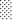) and GE1 amastigotes (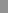) by niranthin from infected macrophages. The number of internalized amastigotes within each infected macrophages were counted under bright field microscope. The results shown are the means of three independent experiments and plotted as mean ± SD. *p < 0.05, **p < 0.01, ***<0.001 (Student's *t*-test) indicate statistical significance between infection control and respective niranthin treatment.Images of Giemsa-stained intracellular parasites in cultured murine macrophages treated with 1, 5 and 10 µM of niranthin compared to an infection control and uninfected control. Arrows indicate the internalized parasites.Comparative analysis of effectiveness of niranthin as combination therapy with SAG on GE1 amastigotes. The results shown are the means of three independent experiments with Standard Deviation. *p < 0.05, **p < 0.01, ***<0.001 (Student's *t*-test). NS denotes there is no significant difference between GE1-infected macrophages and corresponding SAG treatment.Flow cytometric analysis of levels of expression of P-gp in murine peritoneal Mϕ following *in vitro* infection with Sb^R^ *L. donovani* (GE1) and after treatment with niranthin and SAG. Panel I shows the histogram of mean fluorescence intensity. Mean fluorescence intensity was plotted for each sample representing of three independent experiments with Standard Error (panel II). *p < 0.05 (Student's *t*-test). **Indicates significant difference between control macrophages and infection groups or co-treatment groups (*p* < 0.01).

To further investigate the anti-leishmanial activity of niranthin, primary murine macrophages were infected with early passage *L. donovani* AG83 promastigotes (Sb^S^) and SAG-resistant (Sb^R^) GE1 parasites *in vitro* and incubated with different concentrations of niranthin for 24 h. Counting of intracellular amastigotes after treatment showed that niranthin effectively inhibited macrophage infection of both SAG-sensitive and -resistant strains (Fig 5A and B) and the parasite burden was reduced by almost 98% at 25 µM niranthin. The EC_50_ of intracellular parasite clearance was determined using nonlinear curve fitting (Table 2). Also the percentage of infected macrophages was reduced after subsequent treatment with these compounds (Supporting Information Fig S4).

**Table 2:** Effect of niranthin on *Leishmania* amastigotes in macrophages

Parasite name	EC_50_ (µM)
AG83	1.26 ± 0.21
GE1	1.68 ± 0.18

To further assess the effect of niranthin on SAG-resistant parasites, primary macrophages were infected with Sb^R^ (GE1) parasites and treated with 500 nM niranthin (suboptimal concentration) in combination with SAG (0–10 µg/ml) for 24 h and fixed and intracellular amastigotes were counted following Giemsa staining. SAG alone (up to 10 µg/ml) reduced the number of resistant intracellular amastigotes by no more than 32% while co-treatment with the sub-optimal dose of niranthin reduced the parasitic burden by almost 95% (Fig 5C). Thus, the efficacy of SAG can be improved by combination treatment with nirathin.

Overexpression of ABC transporters like permeability glycoprotein (P-gp, also called MDR1) causes antimony resistance in *Leishmania* (Mookerjee et al, 2008). Analysis of mean fluorescence intensity of P-gp showed that the intensity was enhanced by 3.5-fold upon GE1 infection, which on treatment with SAG (5 µg/ml) increased up to 4.4-fold compared to uninfected macrophages (Fig 5D). Upon incubation with 500 nM niranthin, the level of P-gp was reduced by almost 2.6-fold (considering SAG-treated, GE1-infected macrophages as the baseline); although the number of GE1 parasites was only reduced up to 20% by the treatment (Fig 5A). This observation supports that niranthin can affect P-gp levels at suboptimal concentration in infected macrophages. Uninfected normal macrophages poorly express P-gp on their cell surface and thus niranthin has no effect on P-gp expression under these conditions (Supporting Information Fig S5). Co-treatment of 5 µg/ml SAG with 500 nM of niranthin profoundly reduces the parasitic burden almost to that of AG83-infected macrophages (Fig 5C). This result confirms that the inhibition of P-gp expression can be achieved by the natural compound niranthin to prevent Sb efflux and promote SAG-mediated parasite clearance in case of SAG-resistant parasite infection.

### *In vivo* anti-leishmanial efficacy of niranthin in a mouse model of experimental visceral leishmaniasis via induction of iNOS, reactive nitrogen intermediates and reactive oxygen species

**Figure 6:**
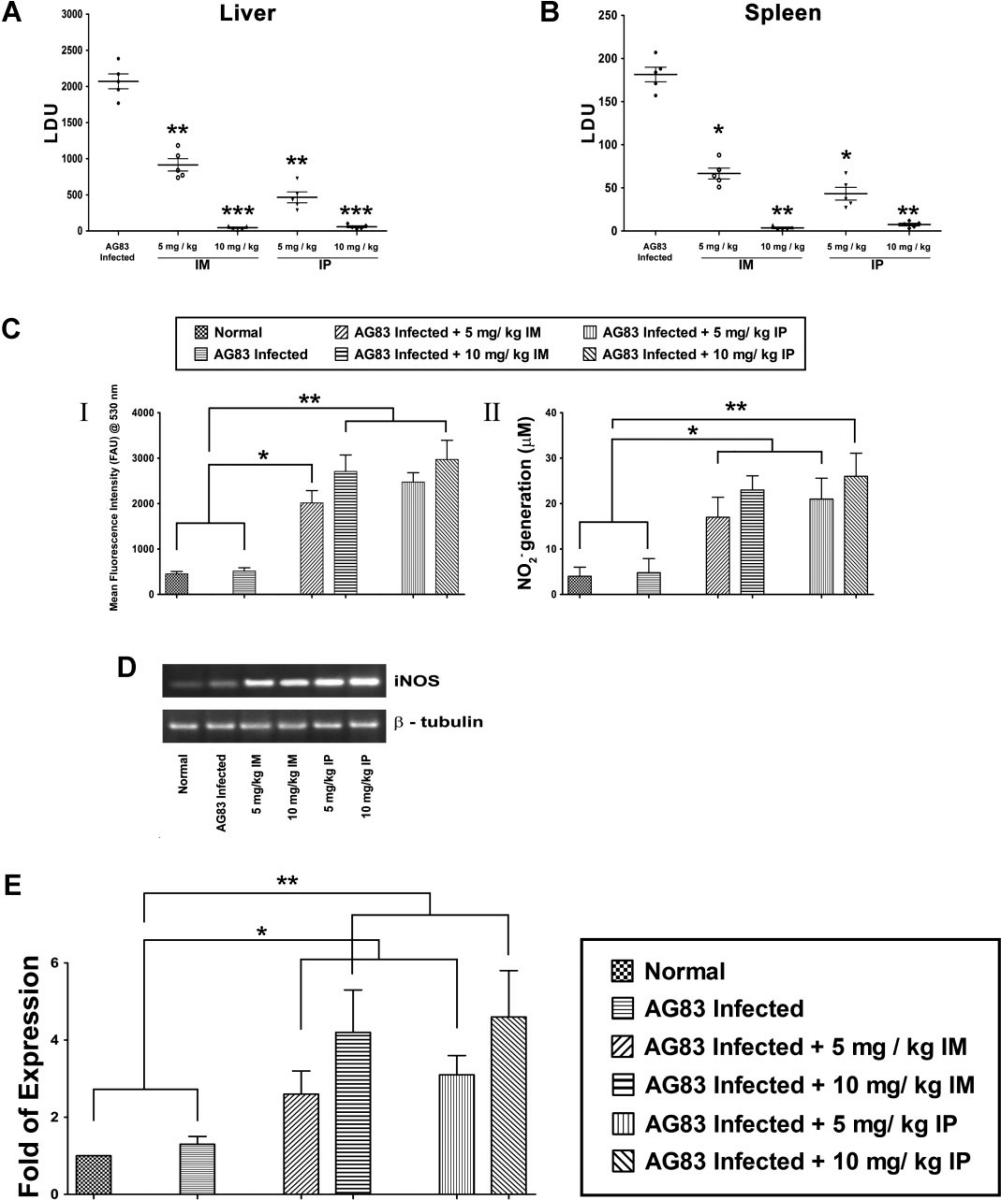
Total parasite burden in BALB/c mice after niranthin treatment **A,B.** *In vivo* leishmanicidal efficacy of niranthin in BALB/c mice infected with AG83 promastigotes via intracardiac route. Niranthin was given at a dosage of 5 and 10 mg/kg/day intramuscularly and intraperitoneally separately, for 3 weeks (2 times per week), starting on day 21 after infection. Animals were sacrificed 10 days after treatment and liver (**A**) and splenic (**B**) parasite load was determined for all groups. Untreated, infected mice were used as controls. Liver and spleen parasite burden were determined by stamp-smear method and expressed as LDU. Data represent mean ± SEM (*n* = 5 mice per group). *p < 0.05, **p < 0.01 and ***p < 0.005 (Student's *t*-test), as compared to different niranthin treatment with infection control.**C.** *In vivo* generation of ROS and NO from infected and cured BSLB/c mice. Following treatment with niranthin (5 and 10 mg/kg/day), splenocytes (2 × 10^6^) from different experimental mice groups were isolated and incubated with SLA for 72 h in 5% CO_2_ incubator at 37°C. ROS generation was measured by H_2_DCFDA probe (**I**) and culture supernatant was used to evaluate NO generation by Griess method (**II**). Results are representative of one of the three experiments. Data presents means ± SD. *p < 0.05, **p < 0.01 (Student's *t*-test).**D,E.** iNOS mRNA expressions (**D**) were evaluated by RT PCR analysis of different experimental groups and fold of expressions (**E**) were calculated using Bio-Rad Quantity One software. mRNA levels were normalized to β-tubulin and expressed as a fold change compared to uninfected control. Data were representative of three experiments and expressed as means ± SD. *p < 0.05, **p < 0.01 (Student's *t*-test), compared to different niranthin treatment either with non-infected or infected groups.

Subversion of macrophage immunomodulatory effect particularly involved in immune surveillance and macrophage activation and suppression of pro-inflammatory cytokine production allow the intracellular parasites to inhibit the host protective response that would otherwise be activated against the organism. Hence, we focused our interests on the *in vivo* animal model of *L. donovani* infection to confirm the *in vitro* leishmanicidal activity of niranthin. BALB/c mice were infected via intracardiac route with *L. donovani* AG83 promastigotes, treated with niranthin (5 and 10 mg/kg/day, intramuscularly and intraperitonealy) and examined 3 weeks post infection, 10 days after the last treatment. During the study, all mice remained healthy and no remarkable change in body weight was observed. At 5 mg/kg/day, there was 56 and 77% reduction of liver parasite burden (Fig 6A) and 63 and 76% (Fig 6B) reduction of splenic parasite load after intramuscular and intraperitoneal niranthin administration, respectively. This inhibition was almost complete using 10 mg/kg/day of niranthin with almost complete suppression of splenic and liver parasite load (Fig 6A and B).

ROS and NO are two pf the most potent macrophage-derived versatile microbicidal players in the immune system. They are involved in the pathogenesis and control of infection of several diseases (Murray & Nathan, 1999). NO is produced in macrophages activated by cytokines, microbial compounds or both, and is derived from l-arginine by the enzymatic activity of inducible NO synthase (iNOS). We therefore investigated the generation of NO in culture supernatants of splenocytes isolated from the different experimental groups above. After stimulation with SLA (100 µg/ml), splenocytes from 5 and 10 mg/kg/day (intramuscularly) niranthin-treated mice showed a 4- and 5.3-fold induction of ROS generation compared with corresponding infected, untreated controls (Fig 6C, panel I). After intraperitoneal injection, a 4.8- and 5.8-fold induction of ROS generation was observed, respectively (Fig 6C, panel I). In a similar fashion, a significant increase in nitrite generation was detected in niranthin-treated mice. Stimulation with SLA produced only 4.8 M nitrite/10^6^ cells, while 15.8 and 23.2 µM/10^6^ cells of nitrite were produced in cells from 5 and 10 mg/kg intramuscularly niranthin-treated mice (Fig 6C, panel II). A similar result was observed for intraperitonealy treated mice (Fig 6C). Consistent with the NO_2_ generation, the levels of iNOS mRNA were significantly upregulated (4.2- and 4.6-fold) after 10 mg/kg/day intramuscular and intraperitoneal injection of the compound, respectively (Fig 6D and E). Collectively, these results suggest that niranthin-treated mice initiate a strong anti-microbial response involving production of NO and ROS and reversing the immunosuppressive state after Leishmania infection towards an active immune response, which potentially accounts for the reduction of parasite burden in the infected BALB/c mice.

### Niranthin induces proliferation of splenocytes, which ultimately augments a host protective Th1 cytokine response

**Figure 7:**
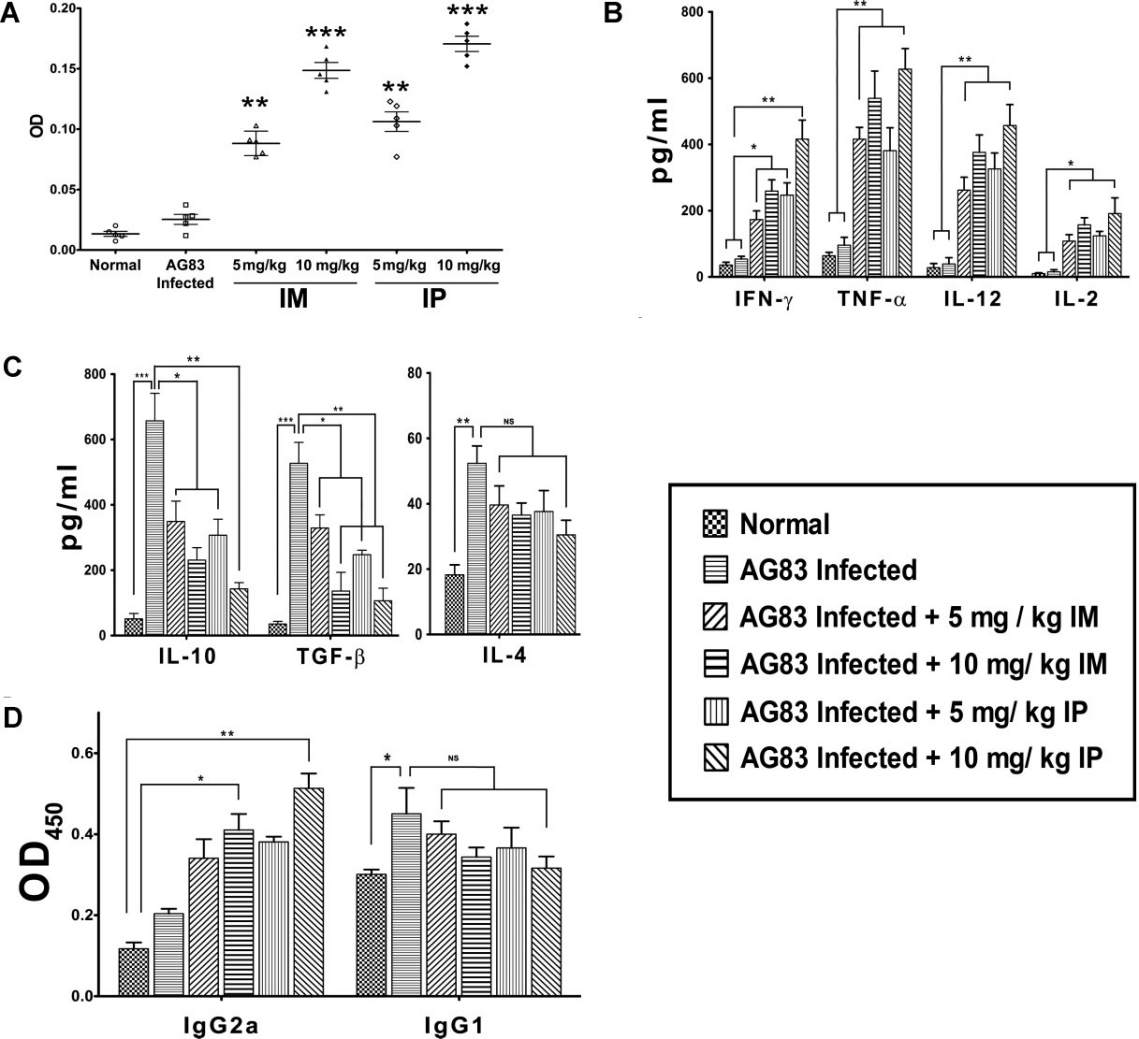
Effect of niranthin on T-cell response and cytokine expression in *L. donovani*-infected mice BALB/c mice were infected with *L. donovani* promastigotes and treated with niranthin as described in Materials and Methods. Ten days post therapy, mice were sacrificed and 5 × 10^5^ splenocytes were incubated with 25 µg/ml SLA in 5% CO_2_ incubator at 37°C for 72 h. T-cell proliferation was measured by MTT assay compared to infected controls. **p < 0.01 and ***p < 0.005 (Student's *t*-test), compared to different niranthin treatment with infected group.Differential pattern of Th1 cytokine production following therapy with nirasnthin in infected BALB/c mice. Splenocytes isolated from AG83-infected mice after indicated treatments were plated aseptically, and stimulated with SLA (25 µg/ml) for 72 h. IFN-γ, TNF-α, IL-12 and IL-2 cytokine levels in supernatants of splenocyte cultures were assayed by ELISA. *p < 0.05, ***p < 0.01 (Student's *t*-test) compared to uninfected or infected mice groups with respective niranthin-treatment.Downregulation of Th2-cytokine expression profile following treatment with niranthin was assayed in culture supernatants of SLA-pulsed splenocytes of different experimental groups as mentioned in Materials and Methods. Values represent the mean ± SD (3–5 mice/group). *p < 0.05, **p < 0.01 (Student's *t*-test). ***p < 0.001 indicates significant difference between uninfected animals and AG83-infected groups. NS indicates differences are not significant.Humoral response associated with niranthin treatment. Sera from untreated and treated mice were analysed individually by ELISA for detection of IgG1 and IgG2a antibodies in different experimental groups of mice (3–5 mice/group). The results are representative of three independent experiments and data represent mean ± SD. *p < 0.05 (Student's *t*-test). NS indicates differences are not significant.

Visceral leishmaniasis is associated with impaired cell-mediated immune responses, which is reflected by marked T-cell anergy specific to *Leishmania* antigens (Carvalho et al, 1994; Gifawesen & Farrell, 1989). Absence of splenic and liver amastigotes with strong NO_2_ generation led us to investigate whether niranthin can facilitate a strong immune response, which is directed against leishmaniasis. In order to verify our assumption, we performed an SLA-specific T-cell proliferation assay in whole splenocytes. In contrast to infected mice, intraperitoneally drug-treated mice (10 mg/kg/day) showed an almost 13-fold higher proliferation of T-cells than the infected control group (Fig 7A). Similarly, a sixfold higher T-cell proliferation was noticed when mice were treated intramuscularly (5 mg/kg/day) with niranthin and T-cell proliferation increased up to 11-fold when mice were treated intramuscularly with 10 mg/kg/day (Fig 7A). To further confirm that the niranthin-mediated proliferative response is due to T cells, pre-treatment with anti-CD4^+^ and anti-CD8^+^ antibodies was performed and significantly reduced the proliferation of T cells (Supporting Information, Fig S6). This also implies that proliferation was contributed to by both subsets of T cells.

To evaluate the type of immunological response associated with niranthin treatment, splenic cytokine expression levels were analysed by ELISA in *L. donovani*-infected mice after treatment with niranthin. A general Th1 dominance in niranthin (10 mg/kg/day)-treated mice was evident from 4.8- and 7-fold higher interferon (IFN-γ) secretion in SLA-pulsed splenocytes after intramuscular and intraperitoneal treatment of infected mice, respectively, as compared to the levels in untreated, AG83-infected mice (Fig 7B). The elevated level of IFN-γ production corresponded with a simultaneous upregulation of TNF-α and IL-12 expression. TNF-α was elevated by 8.6- and 11.2-fold while IL-12 levels were enhanced by 5.6- and 6.2-fold after intramuscular and intraperitoneal (10 mg/kg/day) treatment, respectively (Fig 7B). In experimental visceral leishmaniasis, impairment of IL-2 production in T-cell anergic conditions circumvents the impaired innate immunity (Carvalho et al, 1985; Reiner & Finke, 1983). Hence, we analysed the production of IL-2 in different groups of mice, stimulated with or without SLA (50 µg/ml) for 48 h *ex vivo*. A significantly higher amount of IL-2 in the supernatant was observed in case of cells isolated from treated animals. Intramuscular treatment (10 mg/kg/day) led to 10-fold and intraperitoneal treatment led to 12.7-fold increased IL-2 production compared to the corresponding infected control group (Fig 7B).

Immunosuppresive cytokines play a major role for survival of intracellular parasites in the host. Suppression of these cytokines leads to rapid curing of progressive disease *in vivo* (Ghalib et al, 1993). Niranthin-treated, AG83-infected mice showed about 3.2- and 5-fold decrease in IL-10 production after im and ip treatment (10 mg/kg/day), respectively (Fig 7C). Successful chemotherapy also depends on synchronized downregulation of TGF-β. *Ex vivo* assessment (stimulated by SLA) of TGF-β in treated mice showed a declined expression (3.8- and 5.7-fold after im and ip treatment, respectively) compared to infected mice. However, almost a sustained level of IL-4 production has been noticed in all the experimental groups of mice (Fig 7C). Collectively, these data suggest that niranthin could confer protection against leishmaniasis through lymphocytic proliferation of splenocytes as well as switching the immune balance to the host protective Th1 response *in vivo*.

Humoral immunity refers to host protective antibody production and a subtle Th2 activation and cytokine production is associated with this response. IgG2a levels are mainly dependent on production of IFN-γ and IgG2b inducing factor completely restores a normal IgG1 response induced by IL-4 (Coffman et al, 1993). Thus, IgG2a and IgG1 can be used as surrogate markers for Th1 and Th2 responses. Im treatment with 10 mg/kg/day niranthin led to a 2.1-fold higher SLA-specific IgG2a production compared to respective untreated AG83-infected splenocytes, which is similar in case of ip treatment (2.26-fold) (Fig 7D). The levels of IgG1 are not significantly changed in case of niranthin treatment (Fig 7D), which indirectly correlated with a steady secretion of IL-4 (Fig 7C). But a subtle Th1 response elicits IL-12 production from the lymphocytes, which ensures the protective responses.

## DISCUSSION

In the present study, we have shown that niranthin is a potent growth inhibitor of Leishmania promastigotes (insect gut form) and more deadly amastigotes (human infected form). Induction of different nucleases in the drug-treated parasites ultimately triggers oligonucleosomal fragmentation of genomic DNA and induces apoptotic cell death. Niranthin selectively stabilizes topoisomerase I-mediated protein–DNA complex formation in cell culture. This experiment suggests that due to formation of a topoisomerase I–DNA covalent complex, single-strand breaks in DNA are induced, which ultimately trigger the apoptosis in parasites.

A topoisomerase reaction has three general mechanistic steps (Stewart et al, 1998): (i) binding of the enzyme to the substrate DNA, (ii) cleavage of one strand by a trans-esterification reaction followed by strand rotation (Koster et al, 2005) leading to the change of linking number by one or more than one, and (iii) strand religation and turnover of the enzyme. Like CPT (Champoux, 2001), niranthin also stabilizes topoisomerase I–DNA cleavable complex formation *in vitro*. With stabilization of cleavable complex, niranthin inhibits the subsequent religation step as shown under single turnover conditions and thus hampers DNA relaxation activity and ultimately inhibits parasitic replication and transcription of several house-keeping genes (Supporting Information Fig S7).

*In vitro* results show that the relaxation activity of *L. donovani* topoisomerase IB is inhibited in both simultaneous and preincubation conditions. Enzyme kinetics reveals that the inhibition mode is non-competitive, *i.e.* the drug binds to the enzyme–substrate complex as well as the enzyme alone. Reversible interaction has also been established from the dilution study, the *K*_D_ value being in the range of ∼10^−6^ M for LdTOP1LS (Table 1). Binding details with LdTOP1LS using Job's Plot emphasizes that two molecules of niranthin are binding to one molecule of LdTOP1LS. This led us to investigate whether niranthin can bind to each subunit of the enzyme (*i.e.* LdTOP1L and LdTOP1S) separately. It was found that niranthin binds in equimolar fraction to large and small subunits of the enzyme and the affinity of binding to the small subunit is about twofold greater than to the large subunit (Table 1).

We have shown for the first time that niranthin has a profound effect on the clearance of antimony-sensitive (Sb^S^) and -resistant (Sb^R^) intracellular parasites from cultured macrophages. The reduction of parasitic burden for both the parasites is similar when cells were treated with 10 µM of niranthin for 24 h. In clinical isolates of Sb^R^ parasites, a major problem for treatment with SAG or pentostam is the efflux of drugs due to over expression of P-gp on the surface of infected macrophages (Mookerjee et al, 2008). Our results show that upon co-treatment with a non-toxic (to intracellular *Leishmania*) dosage of niranthin (500 nM) with 5 µg/ml of SAG, intracellular resistant *Leishmania* are cleared effectively (up to 95%) likely by reducing P-gp expression in infected macrophages. Niranthin downregulates the expression of P-gp expression at the transcription level associated with GE1-infection (Supporting Information Fig S8) and thus reverses the multidrug resistance phenomenon. Macrophage viability assays suggest that niranthin has no remarkable cytotoxicity up to 50 µM concentration (inhibiting only 12% of growth; Supporting Information Fig S9). These results suggests that SAG is retained within parasites when cells are co-treated with niranthin.

The anti-leishmanial activity of niranthin was then validated in *L. donovani*-infected BALB/c mice. The limitation in using the mouse model for VL is that it cannot reproduce all the clinicopathological features of progressive human VL. Eight weeks post infection, the infected animal is able to mount a cellular response that restricts the parasite replication. Hamsters mimic the active human disease and immunopathological mechanisms associated with VL; however, the non-availability of immunological tools restricts the study of responses associated with the eliminination of the disease undergoing a successful treatment. Thus, for early infection and parasite replication, the mouse model provides mostly all the immunological features similar to human VL. In case of SAG, the intramuscular route (Thakur & Narayan, 2004) and for glucantime, the intraperitoneal route of delivery are clinically used (Gangneux et al, 1996). Accordingly, both routes were used for administration of niranthin at a dose of 10 mg/kg body weight. Control of progression of leishmaniasis switches the cytokine balance from Th1 to Th2 response (Awasthi et al, 2004; Kemp et al, 1993). Therapy with niranthin mounts polarized Th1 responses with enhanced IFN-γ secretion, which elicits a dominant effect on macrophage microbicidal machinery along with NO production. The synergistic effect of IFN-γ and IL-12 with activation of iNOS and reduced Th2-associated cytokine IL-10 and TGF-β responses was also observed in treated mice. An effective leishmanicidal response against *L. donovani* is dependent on an IL-12-mediated Th1 response leading to induction of IFN-γ production. *Leishmania*, upon entering into the host cell, hampers the initial IL-12 production from Th0 subset by inhibiting cellular signalling cascades. Disease severity in BALB/c mice infected with parasites was associated with significantly hampered antigen-specific T-cell proliferation. Detailed immunological analysis of niranthin-treated mice showed a rescue of T-cell-anergic conditions and persistent IgG1 levels, probably regulated by a stable secretion of IL-4 (Alexander et al, 2000; Basu et al, 2005). This was also associated with increased IgG2a and upregulated IL-12 and IFN-γ production in SLA-pulsed splenocytes. A significant increase in the pro-inflammatory cytokine TNF-α was also observed, which is thought to stimulate IL-12-driven IFN-γ secretion. Strong IL-12-driven IFN-γ and TNF-α expression suggests that these cytokines might be involved in the observed upregulation of NO secretion for providing impressive levels of protection. Both reactive nitrogen and oxygen species play an important role in parasite clearance, as inhibition of either prevents macrophage-mediated killing of parasites (Gantt et al, 2001; Roach et al, 1991). Furthermore, IL-10 and TGF-β, both Th1 suppressive cytokines, were found to be down regulated following treatment with niranthin.

In conclusion, our results show that the lignan niranthin isolated from *P. amarus* is a potent *L. donovani* topoisomerase I poison. Recent emergence of *L. donovani* resistant against commonly used SAG can potentially be treated with this compound as a combination or with nirathin alone. In case of Sb^R^ parasites, niranthin inhibits the upregulation of P-gp, so that antimony is retained within the macrophages and exerts its action by killing the parasites. Since niranthin inhibits LdTOP1LS, multiplication of both wild type and antimony-resistant parasites are inhibited due to cytotoxic DNA breaks generated during replication fork movement inside cells. Promising therapeutic effect and protection against *L. donovani* infection also has been achieved by the combined activity of niranthin, which stabilizes topoisomerase I–DNA covalent complex and thus hampering DNA transactions like replication (Fig 1E) as well as transcription (Supporting Information Fig S7). On the other hand, treatment with niranthin causes switching of immunosuppressive humoral and CD4^+^ cell-mediated responses to a protective Th1 type mounting strong ROS and NO generation. Inhibition of P-gp on the transcription level also multiplies its effect on resistant parasites that are not effectively treatable with SAG. Thus, the pluripotent effect of niranthin is due to its effects on parasite topoisomerase I, host P-gp (in case of Sb^R^ parasite), and host ROS and NO production. Inhibition of topoisomerase thus can stall parasitic (amastigote) replication and acts as one of the primary driver of *in vivo* efficacy. Thus, these immunomodulators potentially offer the potential for a broad spectrum of activity against other infectious diseases. This synergistic approach of immunomodulators gains importance augmenting host immune responses during infectious disease, since resistance to a combination therapy is less likely to occur. Structure–function analysis of niranthin with LdTOP1LS interaction along with modelling studies can be well exploited in developing rational approaches to chemotherapy against leishmaniasis.

## MATERIALS AND METHODS

### Chemicals

Niranthin (Fig 1A) used in the study was isolated from the aerial part of *P. amarus*. The structure of the compound was ascertained by spectroscopic analysis and by super imposable infrared (IR) spectra and undepressed mixed melting point with authentic samples (Murugaiyah & Chan, 2007). Alamar Blue reagent was purchased from Invitrogen Life Technologies. DMSO and CPT were purchased from Sigma chemicals (St. Louis, MO, USA). All drugs were dissolved in 100% DMSO at a concentration of 20 mM and stored at −20°C.

### Bioethics

Balb/c mice, originally obtained from Jackson Laboratories, Bar Harbor, ME and reared in the Institute Animal Facilities, were used for experimental purposes with prior approval of the Animal Ethics Committee. The studies and animal handling were approved by IICB Animal Ethical Committee (Registration no. 147/1999, CPCSEA), registered with Committee for the purpose of Control and Supervision on Experiments on Animals (CPCSEA), Govt. of India.

### Measurement of cell viability

The *L. donovani* AG83 promastigotes or primary macrophages (3.0 × 10^6^ cells/ml) were incubated with three different concentrations of niranthin (5, 10 and 20 µM) for different times (2, 4, 6, 8, 10, 12, 16 and 24 h), after which the viability of promastigotes or cells were measured using Alamar Blue dye. It is a cell permeable non-toxic non-fluorescent active ingredient (blue) that uses the natural reducing power of viable cells to convert resazurin to the fluorescent molecule, resorufin (very bright red). Metabolically active cells convert resazurin to resorufin, thereby generating a quantitative measure of viability and cytotoxicity.

### Measurement of ROS

Intracellular ROS levels were measured in niranthin-treated and untreated parasites. Promastigotes (2 × 10^7^ cells/ml) were treated with 20 µM niranthin for indicated time periods. Parasites treated with 0.5% DMSO served as controls. After different treatments, parasites were washed and resuspended in 500 µl of M199 (without phenol red) and loaded with a cell permeant dye H_2_DCFDA for 1 h (BoseDasgupta et al, 2008). For measurement of ROS in treated mice, splenocytes were charged with SLA (50 µg/ml) for 48 h or left without SLA stimulation, resuspended in RPMI1640 medium and incubated with the above probe (2 µg/ml) for 30 min in the dark. Fluorometric measurements (*λ*_ex_ = 510 nm and *λ*_em_ = 525 nm) were performed in triplicate, and the results were expressed as in the mean fluorescence intensity per 10^6^ cells.

### Double staining with annexinV and PI

Externalization of phosphatidyl serine on the outer membrane of untreated and niranthin-treated promastigotes was measured by binding of FITC-annexinV and PI using an annexinV staining kit (Invitrogen Incorporation Ltd). Flow cytometry was carried out for treated and untreated parasites. The gating was done so that the FL-1 channel denotes the mean intensity of FITC-annexinV, whereas the FL-2 channel denotes the mean intensity of PI. The data represented here are a mean of three experiments.

### DNA fragmentation assay

An estimate of the extent of DNA fragmentation after drug treatments was carried out using the cell death detection ELISA kit (Roche Biochemicals). Promastigote samples (5 × 10^6^ cells/ml) were collected at 2-h intervals and the histone-associated DNA fragments (mononucleosome and oligonucleosome) were detected using the manufacturer's protocol. DNA fragmentation was estimated by spectrophotometric measurement of microtiter plates in a Thermo MULTISCAN EX plate reader at 405 nm.

### Cell cycle analysis

Replication process or DNA synthesis was measured by flow cytometry analysis. Exponentially grown *L. donovani* promastigote cells (2.5 × 10^6^ cells) were treated with niranthin (25 µM) for 2, 4 and 8 h. The cells were then harvested and washed three times with PBS (1X), fixed in 50% ethanol (diluted in 1X PBS) for 4 h. The fixed cells were washed thoroughly, treated with 100 µg/ml RNase A and then suspended in 1 ml of staining solution [1 mg/ml stock solution of PI diluted to 3 µM PI in staining buffer (100 mM Tris, pH 7.4, 150 mM NaCl, 1 mM CaCl_2_, 0.5 mM MgCl_2_, 0.1% Nonidet P-40)]. These samples were incubated for 30 min in the dark at room temperature and were analysed via flow cytometry. The cells (20,000) were analysed from each sample. The percentage of cells in G1, S and G2/M phases of the cell cycle were determined in Becton Dickson flow cytometer and data were processed by BD FACS Scan Software (San Diego, CA).

### Immunoband depletion assay

*Leishmania* cells (2 × 10^7^) were cultured for 12 h at 22°C with or without drugs. Nuclear fractions were isolated as described previously (Sen et al, 2004). Briefly, cells were suspended in hypo-osmotic buffer (10 mM Tris/HCl, pH 7.5, 1 mM EDTA, 0.1 mM EGTA. 1 mM PMSF, 1 mM Benzamidine hydrochloride and 5 mM DTT) and homogenized. The homogenate was centrifuged at 10,000 *g* for 10 min at 4°C. The pellet was washed and used as the source of nuclear fraction. Then the nuclear fractions after lysis with 1% SDS were subjected to SDS–PAGE, and the proteins were electrophoretically transferred on to nitrocellulose membranes. Immunoblotting of immobilized proteins were carried out using a rabbit antibody raised against LdTOP1S (Das et al, 2004) and ATPase domain (43 kDa) of *L. donovani* topoisomerase II (Sengupta et al, 2005).

### Purification and reconstitution of recombinant proteins of topoisomerase I activity

*Escherichia coli* BL21 (DE3)pLysS cells harbouring pET16bLdTOP1L and pET16bLdTOP1S, described previously (Das et al, 2006), were separately induced at OD_600_ = 0.6 with 0.5 mM IPTG (isopropyl β-D-thiogalactoside) at 22°C for 12 h. Cells harvested from 1 L of culture were separately lysed by lysozyme/sonication, and the proteins were purified through Ni-NTA (Ni^2+^-nitriloacetate-agarose column (Qiagen) followed by a phosphocellulose column (P11 cellulose; Whatman) as described previously (Das et al, 2004). Finally, the purified proteins LdTOP1L and LdTOP1S were stored at −70°C. The concentrations of each protein were quantified by Bradford reaction using Bio-Rad Protein Estimation Kit according to the manufacturer's protocol.

Purified LdTOP1L was mixed with purified LdTOP1S at a molar ratio of 1:1 and at a total protein concentration of 0.5 mg/ml in reconstitution buffer [50 mM potassium phosphate, pH7.5, 0.5 mM DTT, 1 mM EDTA, 0.1 mM PMSF and 10% v/v glycerol]. The mixture was dialyzed overnight at 4°C and dialyzed fractions were used for plasmid relaxation activity (Das et al, 2004).

### Plasmid cleavage assay

Cleavage assay was carried out as described (Chowdhury et al, 2011). Briefly, 50 fmol of pHOT1 supercoiled DNA (containing topoisomerase I cleavage site) and 100 fmol of reconstituted LdTOP1LS were incubated in standard reaction mixture (50 µl) containing 50 mM Tris–HCl (pH 7.5), 100 mM KCl, 10 mM MgCl_2_, 0.5 mM DTT, 0.5 mM EDTA and 30 µg/ml BSA in the presence of various concentrations of inhibitors at 37°C for 30 min. The reactions were terminated by adding 1% SDS and 150 µg/ml proteinase K and further incubated for 1 h at 37°C. DNA samples were electrophoresed in 1% agarose gel containing 0.5 µg/ml EtBr to resolve more slowly migrating nicked product (Form II) from the supercoiled molecules (Form I).

### Plasmid relaxation assay

The type I DNA topoisomerases were assayed by decreased mobility of the relaxed isomers of supercoiled pBS (SK^+^) [pBluescript (SK^+^)] DNA in agarose gel. The relaxation assay was carried out as described previously with LdTOP1LS (Das et al, 2006), serially diluted in the relaxation buffer (25 mM Tris–HCl, pH 7.5, 5% glycerol, 0.5 mM DTT, 10 mM MgCl_2_, 50 mM KCl, 25 mM EDTA and 150 µg/ml BSA) and supercoiled plasmid pBS (SK^+^) DNA (85–95% were negatively supercoiled, with remainder being nicked circles). The reconstituted enzyme LdTOP1LS was assayed at 50 mM KCl concentration as described before (Chowdhury et al, 2011). For all kinetic studies, the reaction mixtures containing the buffer and DNA were heated to 37°C before addition of the enzymes. The reactions were rapidly quenched using stop solution and kept on ice. The gels were stained with ethidium bromide (EtBr) (0.5 µg/ml) and the amount of supercoiled monomer DNA band fluorescence were quantified by integration using Gel Doc 2000 under UV illumination (Bio-Rad Quantity One Software), as described previously (Chowdhury et al, 2011). Initial velocities (nM of DNA base-pairs relaxed/min) were calculated using the following equation:


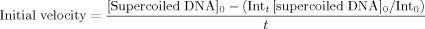


Where ([Supercoiled DNA]_0_ is the initial concentration of supercoiled DNA, Int_0_ is the area under the supercoiled DNA band at zero time and Int_*t*_ is the area at the reaction time *t* (Osheroff et al, 1983). The effect of DNA concentration on the kinetics of relaxation was examined over a range of 8–60 nM supercoiled pBS (SK^+^) DNA (0.16–2.4 µg/25µl of reaction mixture) at constant concentration of 10 mM MgCl_2_ and 0.98 nM enzyme (LdTOP1LS) at 37°C for 1 min. The data were analysed by a Lineweaver–Burk plot. Intercept of the *y*-axis is 1/*V*_max_, and catalytic-centre activity = *V*_max_/enzyme concentration (plasmid molecules relaxed/min/molecule of enzyme).

### Job plot

The binding stoichiometry for each of the inhibitors with LdTOP1LS was determined using the method of continuous variation (Huang, 1982; Ward, 1985). Several mixtures of niranthin and LdTOP1LS were prepared by continuously varying the concentrations of recombinant LdTOP1LS and niranthin in the mixtures keeping the total concentration of inhibitor plus recombinant LdTOP1LS constant at 1.25 µM as described previously (Chowdhury et al, 2011). Reaction mixtures were incubated for 10 min at 25°C and the quenching of tryptophan fluorescence was recorded at 350 nm upon excitation at 295 nm on PerkinElmer LS55 luminescence spectrometer.

### Spectrofluorimetric binding assay

LdTOP1LS (200 nM) was incubated in fluorescence buffer (20 mM Tris/HCl, 50 mM NaCl and 10 mM MgCl_2_) with varying concentrations of niranthin (0–22.5 µM) at 25°C for 10 min as described previously (Chowdhury et al, 2011). Briefly, the fluorescence intensity was measured at 350 nm upon excitation at 295 nm. Excitation and emission slit widths were 2.5 and 5 nm, respectively. The measurements in the fluorescent values (performed in duplicate) in the presence of continuous increasing concentrations of inhibitors were corrected for the inner filter effect for excitation and emission wavelength. The fraction of binding sites (B) occupied by inhibitor was determined by the following equation:


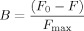


where *F*_0_ is the fluorescence intensity of LdTOP1LS alone in the absence of any inhibitors, *F* is the corrected fluorescence intensity of LdTOP1LS in the presence of inhibitor. *F*_max_ is obtained from the plot of 1/(*F*_0_ − *F*) *versus* 1/[X] and by extrapolating of 1/[X] to zero as shown in Fig 4C, where [X] is the concentration niranthin. The dissociation constant (*K*_D_) was determined as described previously (Acharya et al, 2008) using the following equation:


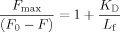


where *L*_f_ denotes the free concentration of inhibitor; *L*_f_ = *C* − *B*[J], where C is the total concentration of inhibitor and [J] is the molar concentration of ligand-binding sites using a stoichiometry from the Job plot.

### *In vitro* macrophage infection

Balb/c mice, originally obtained from Jackson Laboratories, Bar Harbor, ME and reared in the institute animal facilities, were used for experimental purposes with prior approval of the animal ethics committee. Macrophages were isolated from mice 36–48 h post injection (intraperitoneal) with 2% w/v hydrolysed starch by peritoneal lavage with ice-cold phosphate-buffered saline. *In vitro* infection with AG83 and GE1 promastigotes were carried out as described previously (Chowdhury et al, 2011). Niranthin was added at different concentrations (ranging from 0.5 to 50 µM) to infected macrophages and left for another 24 h period. Cells were then fixed in methanol and stained with 2% Giemsa. Percentages of infected cells and total number of intracellular parasites were determined by manual counting in at least 200 cells using light microscope.

**The paper explained**

PROBLEM:

Leishmaniasis is a fatal disease affecting mostly underdeveloped countries worldwide with a high mortality rate and limited treatment options due to the development of drug resistance. Overexpression of surface transporters, like P-glycoprotein (P-gp), associated with resistant parasite infection, effluxes the antimony content from the cell. This restricts the use of commonly used drugs like pentostam or sodium stibogluconate. An alternative treatment strategy with a novel therapeutic target is required for the radical cure of leishmaniasis.

RESULTS:

Our studies define for the first time that niranthin stabilizes the covalent parasite topoisomerase I-DNA complex *in vivo* and *in vitro* and hampers all DNA-related transactions, which ultimately forces the parasite to die. We have determined that niranthin is a promising anti-leishmanial agent against experimental visceral leishmaniasis and a good immunomodulator since niranthin expands the Th1-type immune response with almost sterile protection in mice.

IMPACT:

The major implication of this study is the potential to combat the emergence of drug resistant *Leishmania donovani*. *In vitro* efficacy of downregulation of P-gp on the macrophage surface along with *in vivo* sterile protection provides a possible approach for treatment. Targeting topoisomerase I of the parasite along with a Th2 to Th1 switch provides its dual mode of inhibition. Our studies are expected to open up new avenues for clinical trials with conventional sodium antimony gluconate as a combination therapy as well as parallel treatment.

### Detection of expression of P-gp on macrophage cell surface

The levels of expression of P-gp on infected and treated macrophages or LPS-stimulated macrophages were determined by immunostaining, followed by flow cytometry (Becton Dickson) as described earlier (Mookerjee et al, 2008). The appropriate isotype control was used for each individual case. Since these antibodies were found to recognize MRP1-like molecules in GE1 cells, all stainings were performed 18–24 h after washing the unpermeabilized infected cells in the cold to specifically check the expression of P-gp on the host cell surface.

### Infection of mice and niranthin treatment regimen

For experimental visceral infections, female BALB/c mice (4–6 week old and 20–25 g each) were injected via intracardiac route with 2 × 10^7^ hamster spleen-transformed *L. donovani* promastigotes (suspended in 200 µl of 0.02 M PBS per mouse). Three weeks post infection, niranthin was administered to infected animals via intraperitoneal and intramuscular routes at 5 and 10 mg/kg body weight separately twice a week for a period of 3 weeks. Visceral infection was determined by Giemsa-stained impression smears of spleen and liver from 6-week infected mice and reported as Leishman Donovan Units (LDU), calculated as the number of parasites per 1000 nucleated cells × organ weight (in mg; Stauber, 1956).

### Preparation of soluble leishmanial antigen (SLA)

Soluble Leishmanial antigen (SLA) was prepared from stationary phase *L*. *donovani* AG83 promastigotes as described elsewhere (Saha et al, 1995). Briefly, leishmanial cells (10^9^/ml) were partially lysed by several cycles (minimum six) of freezing (−70°C) and thawing (37°C) followed by 10 min incubation at 4°C. Then it was followed by sonication (thrice for 30 s each) and centrifuged at 12,000 rpm for 40 min at 4°C. The supernatant containing soluble Ag was collected and the protein concentration was determined using Bradford Reagent (PIERCE). Prepared antigen was stored at −70°C until further use.

### Splenocyte proliferation and analysis of different cytokine levels by ELISA

Splenocytes were isolated from different groups of BALB/c mice by mechanical disruption of spleen and lysis of red blood corpuscles (RBC) by 0.14 M Tris buffered NH_4_Cl. Cells were finally suspended in complete medium (RPMI 1640 supplemented with 10% FBS, 100 U/ml penicillin and 100 mg/ml streptomycin and 50 mM β-mercaptoethanol [Sigma–Aldrich]). By direct cell counting using Trypan blue Exclusion method, viable mononuclear cell number was determined. Cells were plated in triplicate at 5 × 10^5^ cells/ml in 96-well plates (BD Biosciences, San Diego, CA) and allowed to proliferate for next 72 h at 37°C in 5% CO_2_ incubator in presence or absence of 10 µg/ml SLA (Sharma & Madhubala, 2001). For *in vitro* depletion study, total splenocytes were incubated with 1 µg/10^6^ cells of anti-CD4^+^ or anti-CD8^+^ mAbs (BD Biosciences) for 2 h at 4°C. Cells were washed thrice for unbound antibodies and cultured in SLA as above. Cells were then treated with MTT (100 µg/ml) solution and kept for next 3 h at 37°C in 5% CO_2_ incubator. The reaction was stopped using 100 µl of stop solution (stock: 4963 µl of isopropanol and 17 µl of concentrated HCl) and kept for 20 min at room temperature. The optical density was taken at A_570_ on an ELISA reader (Multiskan EX; Thermo Fisher Scientific, Waltham, MA). In parallel experiments, cytokine production by SLA (20 µg/ml) pulsed (for 48 h) splenocytes from different mice groups was determined by ELISA kit (BD Biosciences) as per manufacturer's instruction. For IL-12 cytokine analysis, antibody for IL-12 p70 subunit was used for ELISA. TGF-β as secreted in the latent form in the culture supernatants; it needed to be acid-treatment and neutralization for TGF-β measurements.

### Measurement of NO and ROS

Nitric oxide (NO) content in the culture supernatants from SLA pulsed splenocytes cultured for 72 h was monitored by Griess assay method according to Ding et al. (Ding et al, 1988). Briefly, the culture supernatant and the mixture of Greiss reagent [1% sulfanilamide and 0.1% *N*-(1-naphthyl) ethylenediamine dihydrochloride in 2.5% H_3_PO_4_] were mixed at 1:1 ratio and further incubated for 20 min at room temperature, and the OD was determined at 550 nm by ELISA reader (DTX 800 multimode detector, Beckman Coulter). The results were expressed in µM nitrite using NaNO_2_ diluted in culture medium as standard.

### Detection of IgG isotype levels in the serum

Mice were bled 10 days after treatment and sera were prepared and stored at −20°C for further experiments. Antigen-specific serum immunoglobulin Ig-G isotype antibody response was measured by conventional ELISA (BD Biosciences; Banerjee et al, 2008). Briefly, 96-well ELISA plates (BD Biosciences) incubated overnight at 4°C with 2.5 µg/well SLA, were blocked and further incubated with different groups of mice sera (1:2000 dilutions) for 2 h. Plates were washed thoroughly, followed by 1 h incubation at 37°C with peroxidase conjugated goat anti-mouse IgG1 and IgG2a antibodies (BD Pharmingen, San Diego, CA). The plates were then developed for colour reaction with substrate solution (*o*-phenylenediamine dihydrochloride, 0.8 mg/ml in phosphate-citrate buffer (pH 5.0), containing 0.04% H_2_O_2_) for 30 min, and absorbance was measured on ELISA plate reader (DTX 800 Multimode detector, Beckman Coulter) at 450 nm.

### RNA isolation and semi quantitative RT-PCR analysis of cytokines and iNOS

To detect mRNA profile of iNOS, total RNA was isolated from the splenocytes of different mice groups using RNeasy Mini Kit isolation procedure (Qiagen). RNA (2.5 µg) was used as a template for cDNA synthesis. The following forward and reverse primers were used for determination of iNOS expression:

Forward primer: 5′-GCCTCATGCCATTGAGTTCATCAACC-3′
Reverse primer: 5′-GAGCTGTGAATTCCAGAGCCTGAAG-3′

Data for each sample were normalized to β-tubulin mRNA levels and expressed as a fold change with respective controls.

### Statistical analysis

Data are provided as means ± SEM or means ± SD, the number of independent experiments. Data were tested for significance by using the paired Student *t*-test. Differences were considered statistically significant when *P* values were <0.05. Statistical analysis and graphical representation was performed with GraphPad Prism version 5.00 (GraphPad Software, San Diego, CA; USA, http://www.graphpad.com).

For more detailed Materials and Methods see the Supporting Information.

## Supplementary material

Detailed facts of importance to specialist readers are published as ”Supporting Information”. Such documents are peer-reviewed, but not copy-edited or typeset. They are made available as submitted by the authors.
