# Supplementary material for: The lignan niranthin poisons Leishmania donovani topoisomerase IB and favours a Th1 immune response in mice
Source: EMBO Mol Med. 2012 Oct 2;4(10):1126–43. doi: 10.1002/emmm.201201316 (PMC3491841; doi:10.1002/emmm.201201316)
Supplement: Supplementary file 1 [file emmm0004-1126-SD1.pdf]

EMBO
Molecular Medicine

Manuscript EMM-2012-01316

The Lignan Niranthin poisons *Leishmania donovani* topoisomerase IB and favours a Th1 immune response in mice

Sayan Chowdhury, Tulika Mukherjee, Rupkatha Mukhopadhyay, Budhaditya Mukherjee, Souvik Sengupta, Sharmila Chattopadhyay, Parasuraman Jaisankar, Syamal Roy and Hemanta K. Majumder

Corresponding author: Hemanta Majumder, Indian Institute of Chemical Biology

Review timeline:

Submission date:	28 March 2012
Editorial Decision:	10 May 2012
Revision received:	26 June 2012
Editorial Decision:	24 July 2012
Revision received:	30 July 2012
Accepted:	08 August 2012

Transaction Report:

(Note: With the exception of the correction of typographical or spelling errors that could be a source of ambiguity, letters and reports are not edited. The original formatting of letters and referee reports may not be reflected in this compilation.)

1st Editorial Decision 10 May 2012

Thank you for the submission of your manuscript "Niranthin, a Lignan from Phyllanthus amarus, is a Potent Antileishmanial Agent with Mode of Action Targeting to Type IB Topoisomerase of the parasite" to EMBO Molecular Medicine and please accept my apologies for the delayed reply. We have now heard back from the referees whom we asked to evaluate your manuscript. You will see that they find the topic of your manuscript potentially interesting. However, they also raise significant concerns on the study, which should be addressed in a major revision of the manuscript.

In particular, both reviewers highlight that the statistical analysis of the data has to be improved. Importantly, reviewer #1 notes that experiments regarding the in vivo activity of the compound on DNA transactions should be performed and that more insight should be provided on the effect of niranthin on P-gp.

On a more editorial note, we would encourage you to change the title of the manuscript to better reflect its complete content. Please note that EMBO Molecular Medicine does not permit citation of "Data not shown". All data referred to in the paper should be displayed in the main or supplementary figures. "Unpublished observations" may be referred to in exceptional cases, where these are data peripheral to the major message of the paper and are intended to form part of a future or separate study. In addition, please see our Guide to Authors for our guidelines on statistical analysis (http://onlinelibrary.wiley.com/journal/10.1002/(ISSN)1757-4684/homepage/ForAuthors.html#data2).

Given the balance of these evaluations, we feel that we can consider a revision of your manuscript if

you can convincingly address the issues that have been raised within the time constraints outlined below.

Revised manuscripts should be submitted within three months of a request for revision. They will otherwise be treated as new submissions, unless arranged otherwise with the editor.

I look forward to seeing a revised form of your manuscript as soon as possible.

Yours sincerely,

Editor
EMBO Molecular Medicine

***** Reviewer's comments *****

Referee #1 (Comments on Novelty/Model System):

The techyical quality of the manuscript is quoted only medium because statistical analysis are lacking particularly in the analysis of cytokines. If the authors are able to add them , the manuscript will be quotes as high.

Referee #1 (Other Remarks):

The manuscript is well comprehensive and the authors made considerable efforts to render it clear as much as possible for non specialists.
The manuscripts is suitable for publication if the authors can respond to the following minor comments :
- precise the cytokines analysed for IL-12 i.e. Il-12 p40, p35 or p70 and for TGF- beta i.e latent or not
- add statiscal analysis in Figure 6 and 7
- modify refrences 37 and 48.The short tittle of the scientific journal is Infect. Immun. instead of Infect. Immunol.

Referee #2:

Authors identify and characterize niranthin, a lignan, as a Ldtop1B posion. Authors thoroughly investigate the interaction of niranthin with Ldtop1B protein and the Top1B DNA complex in vitro. The authors demonstrate that niranthin kills promastigotes by triggering a PCD-like mechanism. Niranthin also kills intracellular amastigotes, and apparently helps restore sensitivity to drug resistant amastigotes at sub-therapeutic levels. Finally, authors demonstrate that niranthin can reduce parasite burden in an animal model.
The in vitro analysis is thorough and well-performed. The animal experiments are convincing and the authors make the case for niranthin's candidacy as a lead compound.

I have some general and some specific concerns about this manuscript.
General:

I found parts of this manuscript rather hard to read and follow. This is primarily due to imprecise use of language, especially when describing changes. I will provide two specific examples:At the end of the first paragraph of page 23, the authors state "The parasite burden is reduced to almost 98% at 25µM and no visible parasites were seen at 50 µM concentration." A reduction to 98% is no reduction at all. Do the authors mean to say 'by'? Similarly, at the end of page 23, the authors claim that "Upon incubation with 500 nM niranthin, the level of P-gp was greatly reduced to almost 2.6-fold compared to SAG treated macrophages;" What was the baseline for quantifying this reduction? I find this very confusing. This issue arises at multiple places in the manuscript, The authors have put in a lot of effort in to generating the data for this manuscript, and do themselves a disservice by being imprecise in their description of their findings.
There are also sections of this manuscript where the authors' statements need to be backed up by

data. On page 34 in the summarized 'Results', authors state "niranthin stabilizes Topoisomerase I-DNA covalent complex in vivo and in vitro and hampers all DNA related transactions which ultimately forces the cells to die." The authors have only tested the formation of protein-DNA complexes in vivo. Have tests for other DNA transactions - transcription, replication etc. - been performed? If yes, please describe the results in the manuscript as they are important. Likewise on page 26, the authors state "To further confirm that this proliferative response is due to T-cells, pretreatment with anti-CD4+ and anti-CD8+ antibodies significantly reduced (1.63-fold) the generation of T-cells (data not shown). This also implies that proliferation was contributed by both subsets of T-cells." Which dose and regimen was the antibody pre-treatment performed on? Is a 1.63 fold reduction of a 10 fold increase really significant? I would have liked to see more details and data here.

The authors emphasize and elaborate on the interaction of niranthin with Ldtop1B in vitro in their descriptions, but (I think) neglect the other fascinating findings about this molecule's activities. How does niranthin modulate P-gp? Is the effect only on infected macrophages, or can niranthin reduce P-gp expression on uninfected cells? Is this niranthin-mediated increase in sensitivity also presence in parasites not resistant to SAG?
Finally, niranthin is apparently pluripotent in vivo with effects on parasite toposiomerase IB, host P-gp, and host ROS production and iNOS level. Is topo IB poisoning (or inhibition?) the primary driver of in vivo efficacy? A subject worthy of discussion, I would suppose.

Specific questions:

1) Figure 2b western blot. Which experiment is the tubulin blot a control for? Obviously not the topo IB band-depletion since treatments are different. Please insert appropriate control.
2) Why is a KD of 1 μM considered 'weak' binding? Reversible does not equate with weak.
3) What is the logic in selecting very specific data points (out of many options) within each experiment for performing statistical tests?
4) The arrows depicting intracellular amastigotes in figure 5 need to align better. In the small final figure, it is difficult to discern what they are pointing to.
5) The macrophages look different in figure 5 in different drug concentrations. Adherence appears to increase with increasing drug. Why is this? Is this another effect of the compound or a sampling artifact?
6) Were drug doses, regimens and routes chosen empirically? If not, please explain the rationale. It will be helpful to other workers in the field who do animal efficacy studies.
7) I am not sure Figure 8 adds anything to the manuscript.

1st Revision - Authors' Response 26 June 2012

ANSWERS TO REFEREES

We are extremely thankful to the editor and referees for their valued comments. We have addressed all the issues raised by them and have incorporated the corrections in the revised manuscript. We sincerely feel that the revision has improved the quality of the manuscript significantly. The answers to the editor's note and referees' comments are given below.

Editorial note: we would encourage you to change the title of the manuscript to better reflect its complete content. Please note that EMBO Molecular Medicine does not permit citation of "Data not shown". All data referred to in the paper should be displayed in the main or supplementary figures. "Unpublished observations" may be referred to in exceptional cases, where these are data peripheral to the major message of the paper and are intended to form part of a future or separate study.

Answer: We thank the Editor for citing such a valuable issue. For better reflection of our work we have changed the title of the manuscript. The revised title is "Niranthin, a Lignan, intercepts *Leishmania donovani* replication by poisoning topoisomerase IB of parasite and ameliorates Th1 response to cure infected-BALB/c mice".

As EMBO Molecular Medicine does not permit citation of “Data not shown”, we have now put all our data (which were present in previous manuscript as “data not shown”) in the Supplementary Information of the revised manuscript.

When rechecking our previous manuscript, we have found some typographic error. In the revised manuscript we have rectified these issues.

Referee #1 (Comments on Novelty/Model System):

Query 1: The technical quality of the manuscript is quoted only medium because statistical analysis are lacking particularly in the analysis of cytokines. If the authors are able to add them, the manuscript will be quotes as high.

Answer: We thank the reviewer for pointing out this issue. In our revised manuscript we have analysed all the results for statistical significance and added them in our revised figures.

Referee #1 (Other Remarks):

Query 2: Precise the cytokines analysed for IL-12 i.e. Il-12 p40, p35 or p70 and for TGF- beta i.e. latent or not.

Answer: We have analysed IL-12 p70 cytokine and mentioned in the ‘Materials and Methods’ section of the revised manuscript.

The Mouse TGF beta ELISA recognizes the mature/active form of TGF beta 1 without association with Latency Associated Peptide (LAP). So the samples require acid-treatment and neutralization to remove LAP from TGF beta prior to evaluation in this assay. As the supernatant may contain low levels of immune-reactive TGF beta that disassociates from LAP, it is usually acid hydrolysed followed by neutralization for ELISA assay. The above said protocol has been mentioned in ‘Materials and Methods’ Section in the revised manuscript.

Query 3: Add statistical analysis in Figure 6 and 7.

Answer: Statistical analyses have been added for each group of experimental samples and Student’s t-test (paired) have been done and mentioned in the figures and figure legends.

Query 4: Modify references 37 and 48.The short title of the scientific journal is Infect. Immun. instead of Infect. Immunol.

Answer: According to EMBO Molecular Medicine, we have rearranged the Reference list and the modified the error as mentioned by referee in the revised manuscript.

Referee #2: General Comments

Query 1: I found parts of this manuscript rather hard to read and follow. This is primarily due to imprecise use of language, especially when describing changes. I will provide two specific examples: At the end of the first paragraph of page 23, the authors state "The parasite burden is reduced to almost 98% at 25µM and no visible parasites were seen at 50 µM concentration." A reduction to 98% is no reduction at all. Do the authors mean to say 'by'? Similarly, at the end of page 23, the authors claim that "Upon incubation with 500 nM niranthin, the level of P-gp was greatly reduced to almost 2.6-fold compared to SAG treated macrophages;" What was the baseline for quantifying this reduction? I find this very confusing. This issue arises at multiple places in the manuscript; the authors have put in a lot of effort in to generating the data for this manuscript, and do themselves a disservice by being imprecise in their description of their findings.

Answer: We certainly agree with the referee that during describing the changes there are some imprecise language used. As suggested by the referee we have modified the sentences like “The parasite burden is reduced by almost 98% at 25 mM and no visible parasites were seen at 50 mM concentration.” and “Upon incubation with 500 nM niranthin, the level of P-gp was greatly reduced by almost 2.6-fold (considering SAG treated GE1-infected macrophages as baseline); although the clearance of GE1 parasites was only up to 32% (Fig 5A).” In the Results Section the base line also has been mentioned in bracketed phrase. This issue has been resolved in the revised manuscript in Results and Discussion section.

We have carefully gone through the texts of the manuscripts and did a precise description while describing the results.

Query 2: There are also sections of this manuscript where the authors' statements need to be backed up by data. On page 34 in the summarized 'Results', authors state "niranthin stabilizes Topoisomerase I-DNA covalent complex in vivo and in vitro and hampers all DNA related transactions which ultimately forces the cells to die." The authors have only tested the formation of protein-DNA complexes in vivo. Have tests for other DNA transactions - transcription, replication etc. - been performed? If yes, please describe the results in the manuscript as they are important.

Answer: As suggested by the referee we performed cell cycle analysis of niranthin-treated parasites for different time-points which supports for the arrest of replication (Fig 1E). For transcriptional activity, we analysed mRNA expression by RT-PCR of different housekeeping genes for 2, 4, 6 and 8 h. The results are discussed and figure is mentioned as Supplementary information (Fig S7).

Query 3: Likewise on page 26, the authors state "To further confirm that this proliferative response is due to T-cells, pretreatment with anti-CD4+ and anti-CD8+ antibodies significantly reduced (1.63-fold) the generation of T-cells (data not shown). This also implies that proliferation was contributed by both subsets of T-cells." Which dose and regimen was the antibody pre-treatment performed on? Is a 1.63 fold reduction of a 10 fold increase really significant? I would have liked to see more details and data here.

Answer: In the revised manuscript, we mentioned the regimen for anti-CD4+ and anti-CD8+ antibodies in Materials and Methods Section. We have changed the language of reduction. In the revised manuscript we mentioned "To further confirm that this proliferative response is due to T-cells, pretreatment with anti-CD4+ and anti-CD8+ antibodies significantly reduced the generation of T-cells. The level of proliferation being 1.63-fold higher compared to the infected groups without treatment with niranthin." The data are shown in Supplementary Information (Fig S6).

Query 4: The authors emphasize and elaborate on the interaction of niranthin with Ldtop1B in vitro in their descriptions, but (I think) neglect the other fascinating findings about this molecule's activities. How does niranthin modulate P-gp? Is the effect only on infected macrophages, or can niranthin reduce P-gp expression on uninfected cells? Is this niranthin-mediated increase in sensitivity also presence in parasites not resistant to SAG?

Answer: We certainly agree with the referee that we neglected the activity of niranthin on P-gp. So in the revised manuscript we have discussed the fact. We have done Real time PCR analysis of MDR1 gene expression on GE1-infected macrophages followed by niranthin treatment. The results have been discussed and figures are attached in supplementary information (Fig S8).

Macrophages express a basal level of P-glycoprotein on cell surface. Treatment with niranthin has no effect on this basal level. These data are summarized in Discussion section (Fig S5 of Supplementary information).

Only SAG-resistant parasites, on infection, enhance P-gp on cell surface. So in this case treatment with niranthin downregulates MDR1 expression and increases SAG sensitivity. On the other hand, SAG-sensitive parasites do not upregulate MDR1 transporter (Antimicrob Agents Chemother. (2008) 52:1080-93). So niranthin-mediated increase in sensitivity for SAG is not possible in case of SAG-sensitive parasitic infection.

Query 5: Finally, niranthin is apparently pluripotent in vivo with effects on parasite toposiomerase IB, host P-gp, and host ROS production and iNOS level. Is topo IB poisoning (or inhibition?) the primary driver of in vivo efficacy? A subject worthy of discussion, I would suppose.

Answer: Although niranthin is pluripotent, but topoisomerase inhibition leads to cell cycle arrest, which therefore ultimately inhibits parasite multiplication and disease severity. In case of SAG-resistant parasites, SAG can be retained in the macrophages by modulating P-gp expression level at non-toxic condition. Niranthin is an immunomodulator, so it can generate NO and ROS inside cell to clear parasitic load. But the primary driver is topoisomerase I- DNA complex formation as it can limit the amastigote replication inside macrophages. This issue has been discussed in the revised manuscript in Discussion section (Page 23, line 3-6 from top).

Referee #2: Specific questions:

Query 1: Figure 2b western blot. Which experiment is the tubulin blot a control for? Obviously not the topo IB band-depletion since treatments are different. Please insert appropriate control.

Answer: The tubulin blots for each experiments has been performed and Figure 2 has been revised and necessary controls for the experiments has been inserted in the revised manuscript.

Query 2: Why is a KD of 1 µM considered 'weak' binding? Reversible does not equate with weak.

Answer: We surely agree that the reversible interaction does not equate with 'weak' binding. Hence we removed the term and just coined as reversible binding.

Query 3: What is the logic in selecting very specific data points (out of many options) within each experiment for performing statistical tests?

Answer: We have now analysed for almost all the data points for statistical tests. We have analysed the data sets and showed the significant points where p value changes from < 0.05 to < 0.01 or <0.001.

Query 4: The arrows depicting intracellular amastigotes in figure 5 need to align better. In the small final figure, it is difficult to discern what they are pointing to.

Answer: We have now enlarged the Figure 5C and aligned the arrows better for depicting intracellular amastigotes.

Query 5: The macrophages look different in figure 5 in different drug concentrations. Adherence appears to increase with increasing drug. Why is this? Is this another effect of the compound or a sampling artefact?

Answer: We appreciate the point raised by the referee. *Leishmania* infection modulates phagocyte adhesion as early as 2 h post infection and modulation of mononuclear phagocyte adherence to inflamed connective tissue is sustained by infection and modulates adhesion molecules (Infect Immun. (2006) 74:3912-21). After successful therapy the adherence reverts back due to clearance of parasitic loads. We have now included a picture of uninfected macrophages to compare the uninfected, infected and treated cells in Fig 5 (Panel B) of the revised manuscript.

Query 6: Were drug doses, regimens and routes chosen empirically? If not, please explain the rationale. It will be helpful to other workers in the field who do animal efficacy studies.

Answer: The doses and regimens and routes are not chosen empirically. In case of Sodium antimony gluconate (SAG), intramuscular route (Ann Trop Med Parasitol. (2004), 98:129-38) and for glucantime, intraperitoneal routes are used (Antimicrob Agents Chemother. (1996), 40:1214-8). So we worked for both the routes. Both the drugs were given at a dose of 10 mg/kg body weight. So we have chosen the two dosages and the duration of dose is according to SAG treatment. This statement is included in the Discussion section of revised manuscript.

Query 7: I am not sure Figure 8 adds anything to the manuscript.

Answer: As suggested by the referee, the Figure 8 has been removed from main figure and the text.

2nd Editorial Decision 24 July 2012

Thank you for the submission of your revised manuscript to EMBO Molecular Medicine. We have now received the enclosed reports from the referees that were asked to re-assess it. As you will see the reviewer is now globally supportive and I am pleased to inform you that we will be able to accept your manuscript pending the following final amendments:

- Please reply to the referee's comments and adjust the text accordingly.

- We appreciate that you have modified the title of your manuscript. However, we still feel that it remains a bit too long. May I suggest something along the line of: "The lignan Niranthin poisons Leishmania domovani topoisomerase IB and favors a Th1 immune response in mice"?

- We also noted some language incoherences in the text. Would you be so inclined to have a colleague native english speaker look at the main text a last time? Some sentences are still difficult to understand and we feel that the manuscript will gain in readability. Alternatively, we can provide you with a list of potential professional copy-editing services. Please do not hesitate to ask.

© EMBO

- Please provide up to 5 keywords

- We noticed that "Measurement of ROS" happened twice in the Materials and Methods. We would suggest to join both paragraphs into one.

- The last sentence in the Acknowledgements section seems to be unfinished.

Please submit your revised manuscript within two weeks. I look forward to seeing a revised form of your manuscript as soon as possible.

Yours sincerely,

Editor
EMBO Molecular Medicine

***** Reviewer's comments *****

Referee #2:

Two minor points and a comment:
1) Please expand CPT at first use, and then abbreviate thereafter.
2) The description, in the results section, of figure S6 is still very confusing.

I am still not convinced that poisoning via blocking religation is the primary effect of niranthin on toposiomerase IB. According to me, the interaction of niranthin and topo IB is more complicated.

However, since the authors have, in good faith, addressed all the questions raised in the previous review, I consider the manuscript suitable for publication.

2nd Revision - Authors' Response 30 July 2012

ANSWERS TO EDITORIAL NOTE

We are extremely thankful to the editor and referees for their valued comments. We have addressed all the issues raised by them and have incorporated the corrections in the revised manuscript. We sincerely feel that the revision has improved the quality of the manuscript significantly. The answers to the editor's note and referees' comments are given below.

Query 1: Please reply to the referee's comments and adjust the text accordingly.

Answer: We thank the referee for pointing out minor mistakes in the manuscript. We have replied the referee's comment in the Annexure-I and adjusted the text of the as suggested in the revised manuscript.

Query 2: We appreciate that you have modified the title of your manuscript. However, we still feel that it remains a bit too long. May I suggest something along the line of: "The lignan Niranthin poisons Leishmania donovani topoisomerase IB and favours a Th1 immune response in mice"?

Answer: We accept the Editor's suggestion and we have changed the title.

Query 3: We also noted some language incoherences in the text. Would you be so inclined to have a colleague native English speaker look at the main text a last time? Some sentences are still difficult to understand and we feel that the manuscript will gain in readability. Alternatively, we can provide you with a list of potential professional copy-editing services. Please do not hesitate to ask.

Answer: This manuscript has now been examined again by an expert in English. However if the writing is still not satisfactory, I request the Editor to kindly get the manuscript examined by the editing services.

Query 4: Please provide up to 5 keywords.

Answer: The keywords have been provided on submission (Topoisomerase, Leishmania donovani, Chemotherapy, Niranthin).

Query 5: We noticed that "Measurement of ROS" happened twice in the Materials and Methods. We would suggest joining both paragraphs into one.

Answer: We appreciate the editorial board for thorough reading of the manuscript. We now joined the "Measurements of ROS" into a single paragraph in our revised manuscript.

Query 6: The last sentence in the Acknowledgements section seems to be unfinished.

Answer: The sentence has been corrected in the revised manuscript.

Annexure - I

Answers to Referee's Comment

Referee #2:

Query 1: Please expand CPT at first use, and then abbreviate thereafter.

Answer: We expanded CPT as Camptothecin in the Results section and marked in red and then used the abbreviated form thereafter.

Query 2: The description, in the results section, of figure S6 is still very confusing.

Answer: We agree with the reviewer that the statement is confusing. So in order to avoid in understanding, we simplified the text as mentioned below. "To further confirm that niranthin-mediated proliferative response is due to T-cells, pre-treatment with anti-CD4^{+} and anti-CD8^{+} antibodies significantly reduced the generation of T-cells (Fig S6). This also implies that proliferation was contributed by both subsets of T-cells." We have also removed the sentence "The level of proliferation being 1.63-fold higher compared to the infected groups without treatment with niranthin (Fig S6)."

Comment: I am still not convinced that poisoning via blocking religation is the primary effect of niranthin on toposiomerase IB. According to me, the interaction of niranthin and topo IB is more complicated.

Answer: In the third paragraph of Discussion section, we have mentioned that "With stabilization of cleavable complex, niranthin inhibits subsequent religation step as shown in single turnover condition and thus hampers DNA relaxation activity and ultimately inhibits parasitic replication and transcription of several house-keeping genes (Fig S7)." I agree with the reviewer that the mechanism of interaction of niranthin with topoisomerase I and its eventual inhibition is not very simple. Here we have only mentioned the statements based on our experimental study. There may be other mechanisms which we have not investigated in the present study.

© EMBO
