# Supplementary material for: The lignan niranthin poisons Leishmania donovani topoisomerase IB and favours a Th1 immune response in mice
Source: EMBO Mol Med. 2012 Oct 2;4(10):1126–43. doi: 10.1002/emmm.201201316 (PMC3491841; doi:10.1002/emmm.201201316)
Supplement: Supplementary file 2 [file emmm0004-1126-SD2.pdf]

Supplementary Information

Table of Contents

(1) Supplementary Experimental Procedures .... **1 – 3**

(2) Supplementary Figure Legends .... **4 – 7**

(3) Supplementary Figures

- **a. Figure S1:** Measurement of ROS generation **8**
- **b. Figure S2:** Duplex oligonucleotides cleavage assay **9**
- **c. Figure S3:** Religation experiment **10**
- **d. Figure S4:** Percentage of infected macrophages **11**
- **e. Figure S5:** MDR1 expression in uninfected cell **12**
- **f. Figure S6:** Splenocyte proliferation depletion assay **13**
- **g. Figure S7:** Transcription of house-keeping genes **14**
- **h. Figure S8:** Real time analysis of MDR1 gene **15**
- **i. Figure S9:** Macrophage viability assay **16**

Supplementary Experimental Procedures

Analysis of duplex oligonucleotides cleavage assay

The 25-mer duplex of oligonucleotide 1 (5'-GAAAAAAGACTT↓AGAAAAATTTTTA-3') and oligonucleotide 2 (5'-TAAAAATTTTTCTAAGTCTTTTTTC-3') containing a topoisomerase I binding motif was labelled and annealed as described previously (Das et al, 2006). Cleavage was carried out using 20-fold molar excess of the wild-type (LdTOP1LS) enzyme over duplex 25-mer DNA (enzyme, 0.2 μM; DNA, 10 nM). The reactions were carried out in standard assay mixture containing 10 mM Tris–HCl, pH 7.5, 10 mM $MgCl_2$, 0.5 mM EDTA and 50 mM KCl in the presence or absence of drugs at 37°C for 30 min or at indicated time periods. All the reactions were stopped by addition of SDS to a final concentration of 2% (w/v). Samples were precipitated with ethanol, digested with 5 μl of 1 mg/ml trypsin and analyzed by 12% denaturing polyacrylamide gel followed by autoradiography. The amount of strand cleavage in the presence of drugs for the wild-type and mutant enzymes were determined by film densitometry as described previously (Das et al, 2006).

Single turnover cleavage and religation experiment

A 14-mer (5'-GAAAAAAGACTT↓AG-3') oligonucleotide containing topoisomerase IB specific cleavage site was 5'-^{32}P-end labeled and annealed to 25-mer (3'-CTTTTTTCTGAATCTTTTTAAAAAT *p*-5') oligonucleotide as described previously (Das et al, 2006). The suicidal cleavage reaction was carried out with 5 nM of DNA substrate and 0.15 μM of enzyme (LdTOP1LS) in 20 μl reaction mixture under standard assay condition (10 mM Tris–HCl, pH 7.5, 10 mM $MgCl_2$, 0.5 mM EDTA and 50 mM KCl) at 23°C for 4 h in the presence or absence of drugs as described previously (Das et al, 2006). For religation experiments, covalent

complexes generated by incubating suicide DNA substrate with LdTOP1LS in the presence or absence of drugs were transferred to 37°C and pre-incubated for 2 min. Religation was initiated by the addition of 300-fold molar excess of the 11mer religation acceptor oligonucleotides (5 '-OH -AGAAAAATTTT- 3') in the same reaction mixture and incubated for indicated time periods. Finally, all the reactions were stopped by adding SDS, and DNAs were subsequently precipitated by ethanol. Samples were digested with 5 μl of 1mg/ml trypsin, electrophoresed in 12% denaturing polyacrylamide gel and autoradiographed as described previously (Das et al, 2006).

Real – Time PCR analysis

Total RNA was prepared from untreated and treated macrophages using the Total RNA isolation kit (Roche Biochemicals). cDNA was synthesized from 2 μg of total RNA using Superscript II $RNaseH^{-}$ reverse transcriptase (Invitrogen) and $oligo(dT)_{12\text{-}18}$ primers (Invitrogen) according to the manufacturer's instructions. The primers for MDR1 gene were designed such that each set amplified a 280-bp fragment. Three separate reactions were carried out using three different RNA preparations in a 25-μl volume using SYBR Green SuperMix (Applied Biosystems) and the same primer sets in a 7300 real-time PCR system (Applied Biosystems). Reactions were carried out with the following profile: initial denaturation at 95°C for 5 min, followed by 40 cycles with denaturation at 95°C for 60 s, annealing at 55°C for 40 s, and extension at 72°C for 60 s. The PCR was followed by a melt curve analysis to ascertain that the expected products were amplified. The relative amounts of PCR products generated were obtained from the threshold cycle (C_T). The relative amount of the GAPDH gene, used as a control. The level of expression was calculated as:

$$2^{-\Delta\Delta CT} = 2^{-[(CT_{Target} - CT_{Endo})\_experimental - (CT_{Target} - CT_{Endo})\_ control]}$$

Analysis of transcription levels by semiquantitative RT-PCR of house-keeping genes

Parasites were treated with 20 μM of niranthin for 2, 4, 6 and 8 h respectively and semi-quantitative PCR was performed from the above said RNA preparation procedure in 25μl volume using 20 pM each of sense and antisense primers corresponding to GAPDH, α-Tubulin and Ribosomal S8 protein using the following profile: initial denaturation at 95°C for 5 min followed by 25 cycles with denaturation at 95°C for 1 min, annealing at 55°C for 40 s and extension at 68°C for 40 s and a final extension of 3 min.

Supplementary Figure Legends

Figure S1: Measurement of ROS generation for the promastigotes treated with 0.2% DMSO (▼) or 10 μM (♦), 20 μM (▲) niranthin alone or after pre-incubation with NAC (●). After incubation with H_2DCFDA the fluorescence intensity was measured at 530 nm. Values were taken in triplicate, averaged and plotted against time. Data represent means ± S.E. (n=3).

Figure S2: Graphical representation of the extent of covalent complex formation plotted in the plasmid cleavage assay (Figure 2C) as increasing concentrations of niranthin. **(B)** Duplex oligonucleotide substrate with topoisomerase IB cleavage site. **(C)** Duplex oligonucleotide DNA cleavage induced by niranthin and CPT. Cleavage reactions and electrophoresis in a denaturating polyacrylamide gel were performed as described in Supplemental Experimental Procedures. Lane 1, 10 nM of 5'-^{32}P-end labeled 25mer duplex oligonucleotides as indicated above. Lane 2, same as lane 1, but incubated with 0.2 μM of LdTOP1LS in the absence of inhibitor. Lane 3, same as lane 2, but incubated with 50 μM of CPT, for 30 min at 37°C. Lanes 4-6, same as lane 2, but incubated with 5, 10 and 25 μM of niranthin for 30 min at 37°C. Positions of uncleaved oligonucleotide (25-mer) and the cleavage product (12-mer oligonucleotide complexed with residual topo I) are indicated. All the reactions were stopped by addition of SDS to a final concentration of 2% (w/v). Samples were precipitated with ethanol, digested with trypsin and analyzed by 12% denaturing polyacrylamide gel as described in Experimental Procedures. The results depicted were performed three times and representative data are from one set of these experiments.

Figure S3: Suicidal cleavage assay was performed with the 5'-^{32}P-end-labeled suicide DNA substrate (14-mer/25-mer) as indicated. The DNA substrate was incubated with LdTOP1LS in

the presence and in the absence of inhibitors at 23°C as described in the Supplementary Experimental Procedures. Active cleavage complexes containing LdTOP1LS attached to the covalently cleaved 12-mer of the suicide substrate were made to react with 5'-hydroxyl-terminated 11-mer to form a 23-mer product for 10 min at 37°C, and the products were analysed using a denaturing polyacrylamide gel. Lane 1, suicide DNA substrate only; lane 2, same as lane 1, but in the presence of LdTOP1LS and 5'-hydroxyl-terminated 11-mer religation oligonucleotides; lane 3, same as lane 2, but with 50 μM CPT added simultaneously with the enzyme and DNA in the suicidal cleavage assay; lanes 4 and 5, same as lane 2, but with 25 and 50 μM of niranthin added simultaneously with the enzyme and DNA in the suicidal cleavage assay respectively; lane 6, same as lane 2, but with 20 μM DHBA pre-incubated for 5 min at 23°C with the enzyme before the addition of DNA and CPT in the suicidal cleavage assay; lane 7 and 8, same as lane 5, but followed by addition of 25 and 50 μM niranthin simultaneously in the cleavage assay respectively; lane 9, same as lane 2, but 50 μM CPT was added after suicidal cleavage reaction (on enzyme–substrate complex) together with 11-mer religation oligonucleotide; lanes 10-11, same as lane 2, but 25 and 50 μM of niranthin were added after suicidal cleavage reaction (on enzyme–substrate complex) together with 11-mer religation oligonucleotide respectively. All the reactions were stopped by addition of 2% (w/v) SDS. The samples were precipitated with ethanol, digested with trypsin and analysed by denaturing polyacrylamide sequencing gel electrophoresis. The uncleaved suicidal oligonucleotide, covalent complex and the religation products are indicated.

Figure S4: Total number of infected macrophages after niranthin treatment. Dose dependent decrease of macrophages infected either with AG83 amastigotes ( ) or GE1 amastigotes ( ) by niranthin in mouse peritoneal macrophages. The number of internalized

amastigotes within each infected macrophages were counted under bright field microscope. The results shown are the means of three independent experiments and plotted as mean ± S.D. * < 0.05, ** < 0.01 (Student's t-test). *** indicates significant difference between GE1 infection control and 25 μM niranthin treatments (P < 0.001).

Figure S5: Effect of niranthin on uninfected macrophages. Flow cytometric analysis of levels of expression of P-gp in murine peritoneal Mϕ following treatment with niranthin. Panel I shows the histogram of mean fluorescence intensity. Mean fluorescence intensity was plotted for each sample representing of three independent experiments with Standard Deviation (panel II). NS denotes the data are non-significant.

Figure S6: Effect of $CD4^+$ and $CD8^+$ cells on niranthin-induced T-cell proliferation. Total, $CD4^+$ and $CD8^+$ T-cell-depleted splenocytes from niranthin-administered groups of mice were stimulated in vitro with SLA (20 mg/ml). After 48 h, MTT asay was performed as described in 'Materials and Methods'. Data represent mean ± SD (5 mice/ group) in triplicates. * < 0.05, ** p < 0.01 and *** p < 0.001.

Figure S7: Gene expression of GAPDH, α-tubulin and ribosomal S8 protein were carried out in DMSO-treated or niranthin-treated (20 μM for 2, 4, 6 and 8 h) parasites by RT-PCR. RT-PCR was carried out for 25 cycles using RNA isolated from the above stated parasites and the products were run in 1.5% agarose gel accordingly.

Figure S8: MDR1 gene expression analysis in Sb^R-infected macrophages treated with or without niranthin. mRNA expression levels in normal, Sb^R-infected, SAG-treated Sb^R-infected, niranthin-treated Sb^R-infected and niranthin-treated Sb^R-infected (SAG-treated) macrophages were determined by real-time RT-PCR, carried out for 40 cycles using RNA

isolated from these different groups. Fold of expression was calculated from their respective *CT* values using the formula as described in 'Supplementary Materials and Methods' and expressed accordingly. Data represents mean ± S.D. (n=3). * = $p< 0.05$ (Student's t-test), compared to GE1-infected macrophages with the normal uninfected macrophages and ** $p< 0.01$ (Student's t-test), compared to niranthin treated GE1-infected (SAG-treated) macrophages with niranthin-treated respective macrophages.

Figure S9: Effect of niranthin on primary mouse peritoneal macrophages. Primary mouse macrophages isolated as described in 'Materials and Methods' were cultured in the presence of 0.2% DMSO or 50 μM of niranthin for 24 h. Percentage of viable macrophages was measured by Alamar Blue reagent. Values were taken in triplicate, averaged and plotted against time. Data represent means ± S.D. (n=3). * $p < 0.05$, as compared to 50 μM niranthin treatment.

Figure S1

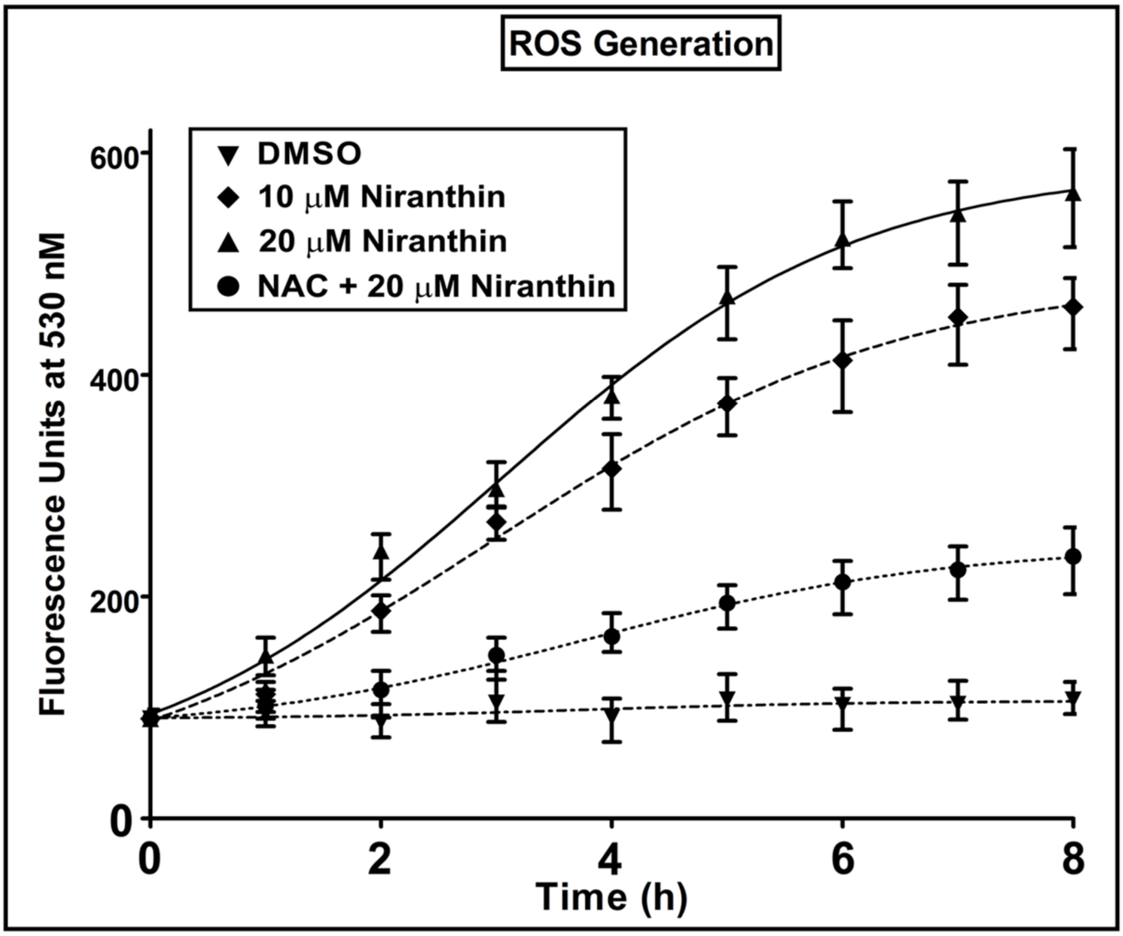
ROS Generation
DMSO
10 μM Niranthin
20 μM Niranthin
NAC + 20 μM Niranthin
Fluorescence Units at 530 nM
600
400
200
0
0
2
4
6
8
Time (h)

Figure S2

A

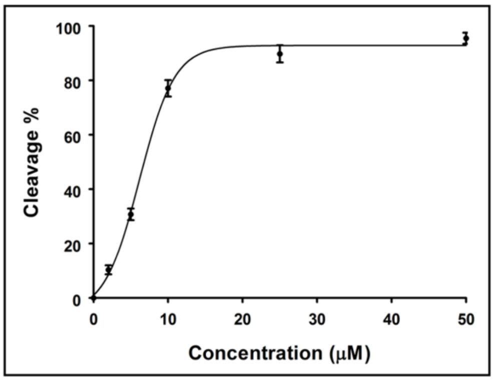

B

Oligo 1: 5' ^{32}P- GAAAAAAGACTT↓AGAAAAATTTTTA 3'
Oligo 2: 3' CTTTTTTCTGAA TCTTTTTAAAAAT 5'

C

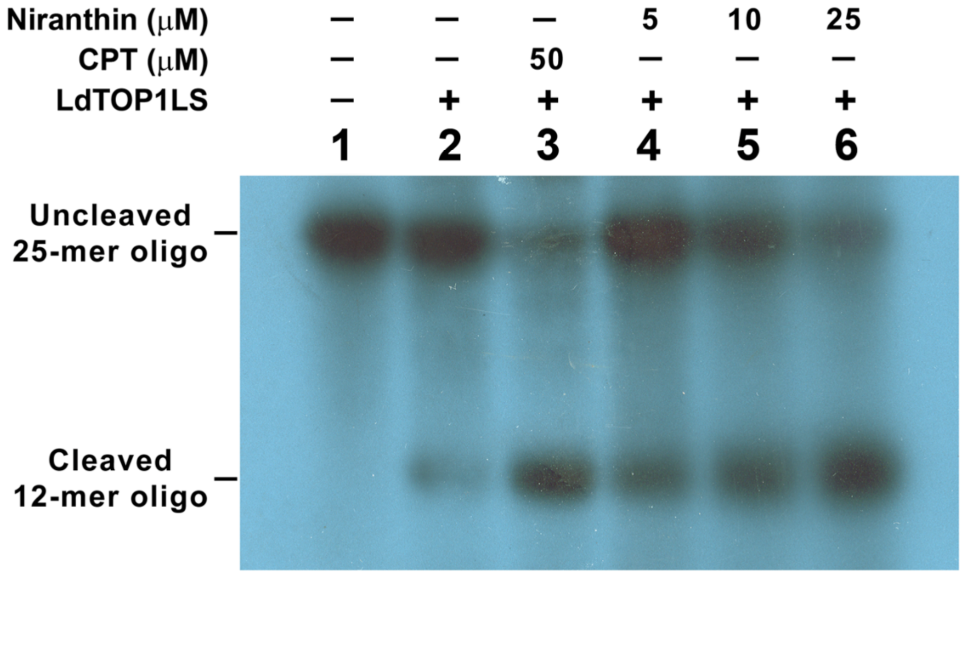

Figure S3

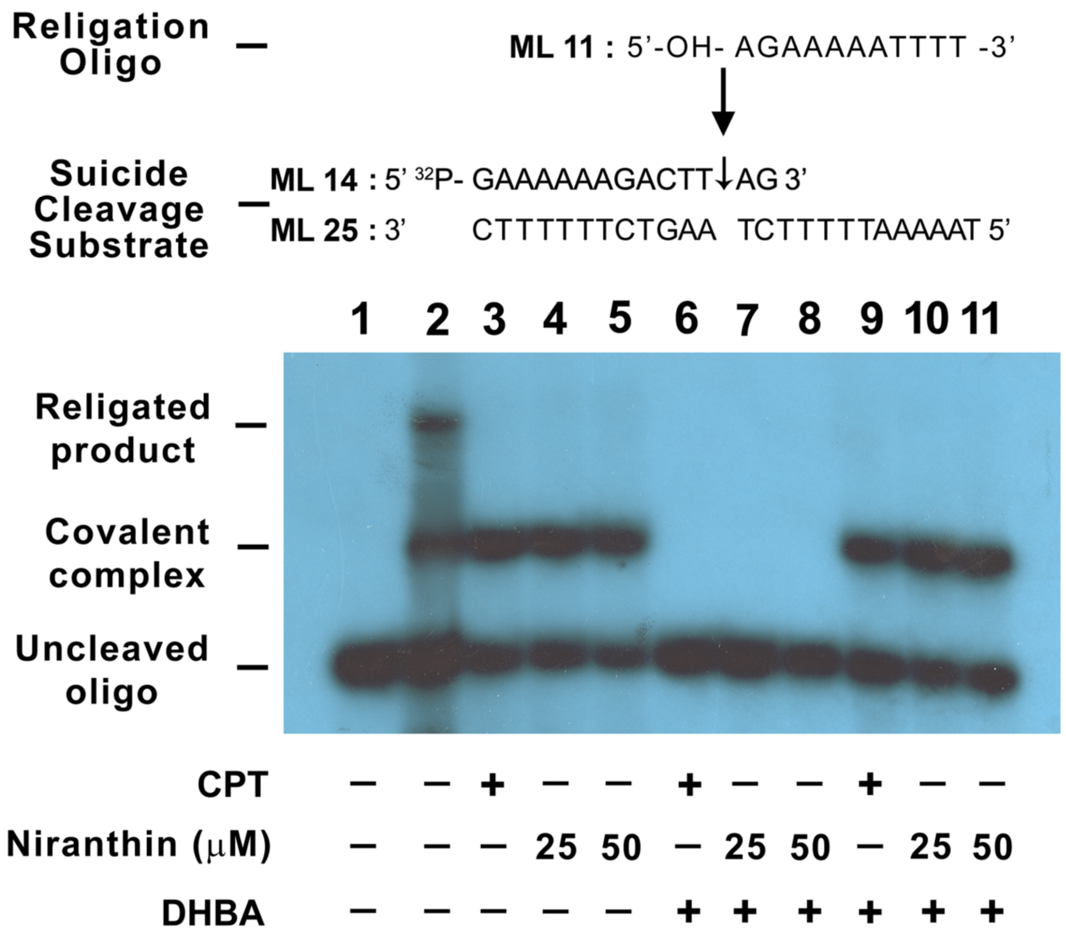
Religation Oligo
ML 11 : 5'-OH- AGAAAAATTTT -3'
Suicide Cleavage Substrate
ML 14 : 5' 32P- GAAAAAAGACTT↓AG 3'
ML 25 : 3' CTTTTTTCTGAA TCTTTTTAAAAAT 5'
1 2 3 4 5 6 7 8 9 10 11
Religated product
Covalent complex
Uncleaved oligo
CPT – – + – – + – – + – –
Niranthin (μM) – – – 25 50 – 25 50 – 25 50
DHBA – – – – – + + + + + +

Figure S4

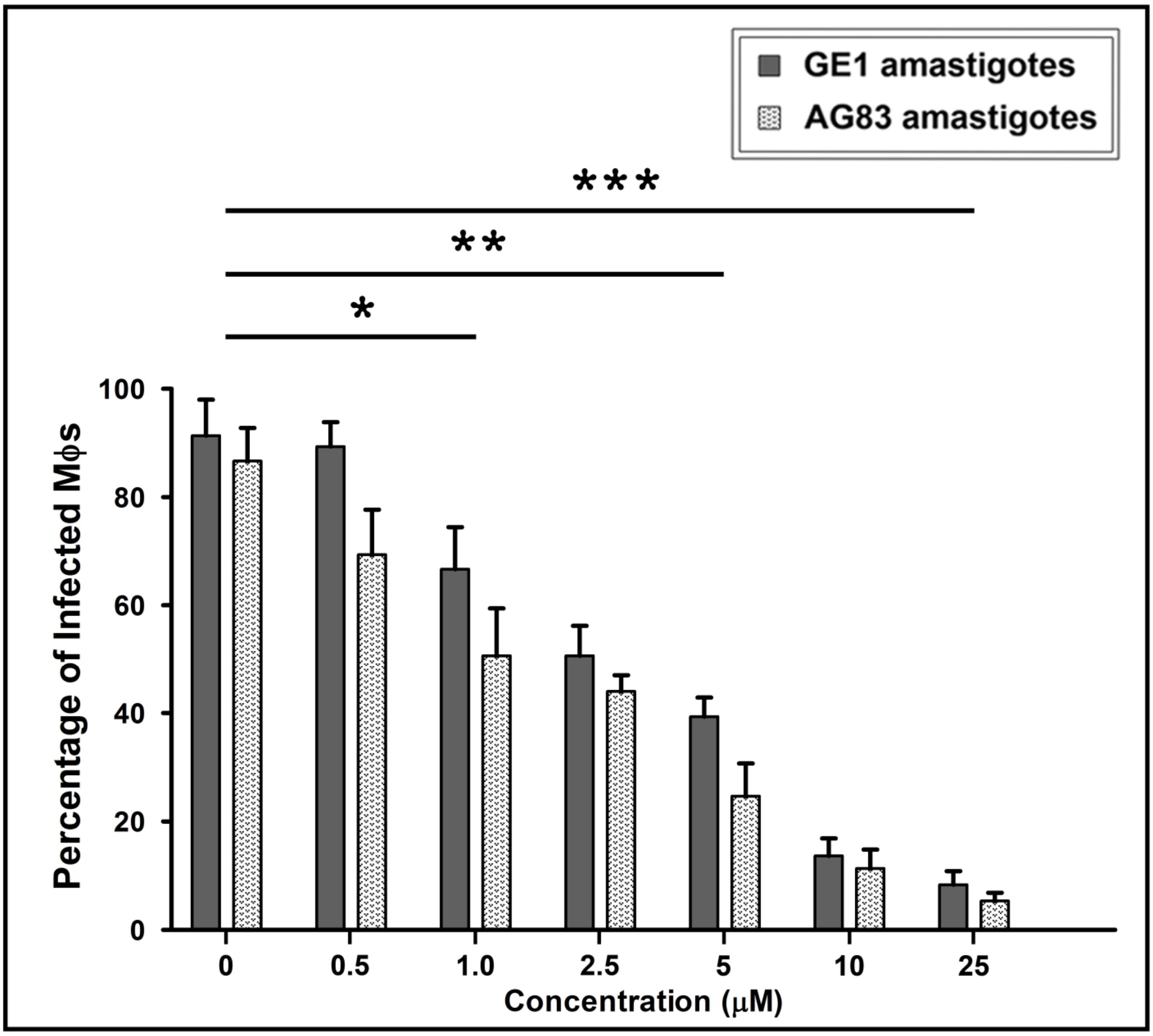
GE1 amastigotes
AG83 amastigotes
***
**
*
Percentage of Infected Mϕs
100
80
60
40
20
0
0
0.5
1.0
2.5
5
10
25
Concentration (μM)

Figure S5

I

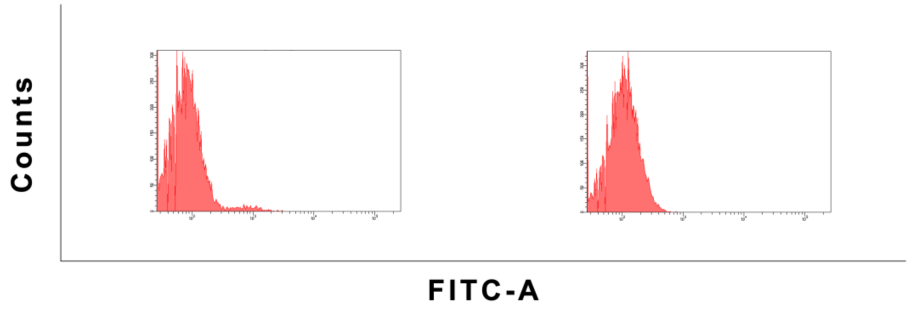

II

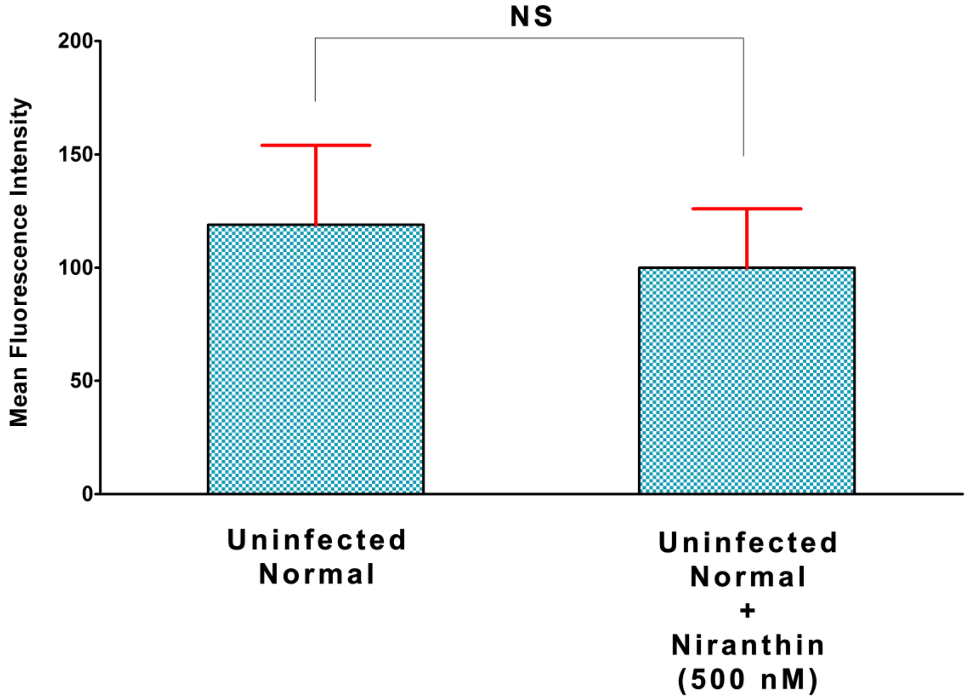

Figure S6

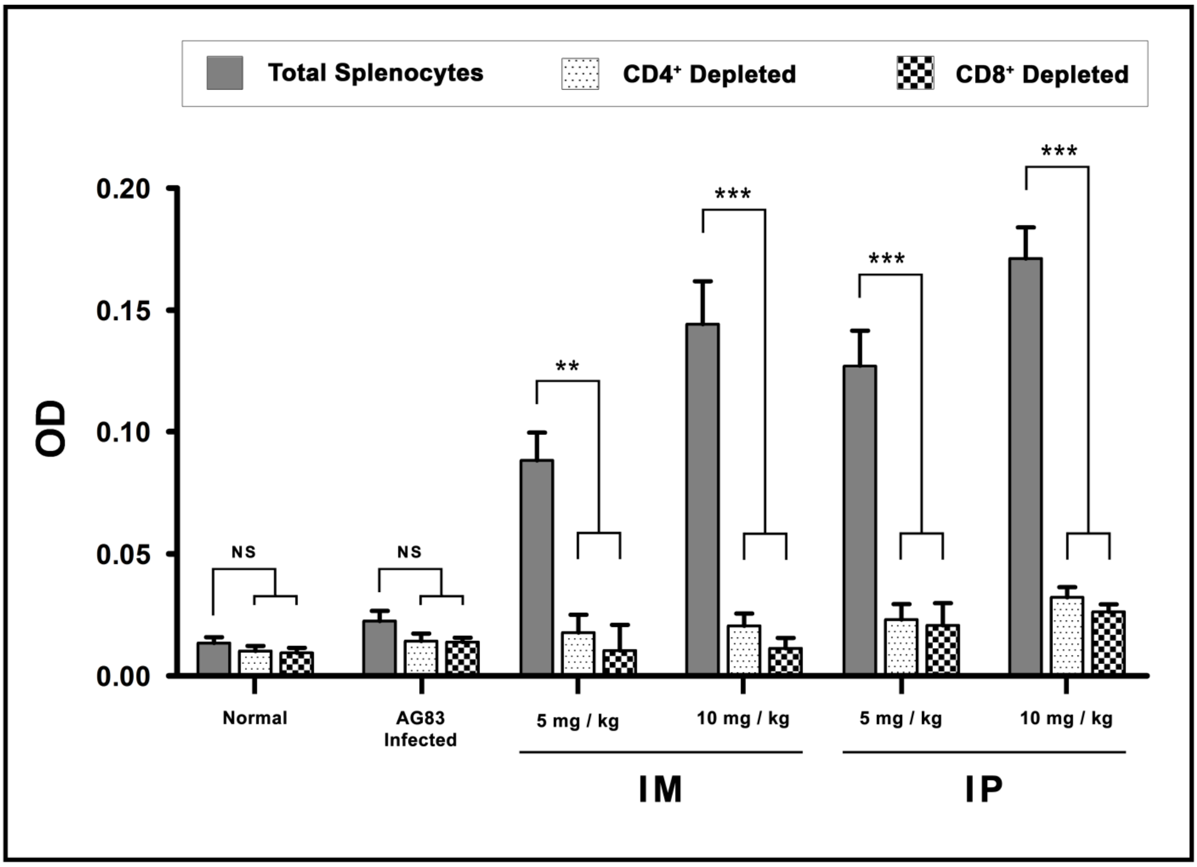

Total Splenocytes
CD4⁺ Depleted
CD8⁺ Depleted
0.20
0.15
0.10
0.05
0.00
OD
NS
NS
**
***
***
***
Normal
AG83 Infected
5 mg / kg
10 mg / kg
5 mg / kg
10 mg / kg
IM
IP

Figure S7

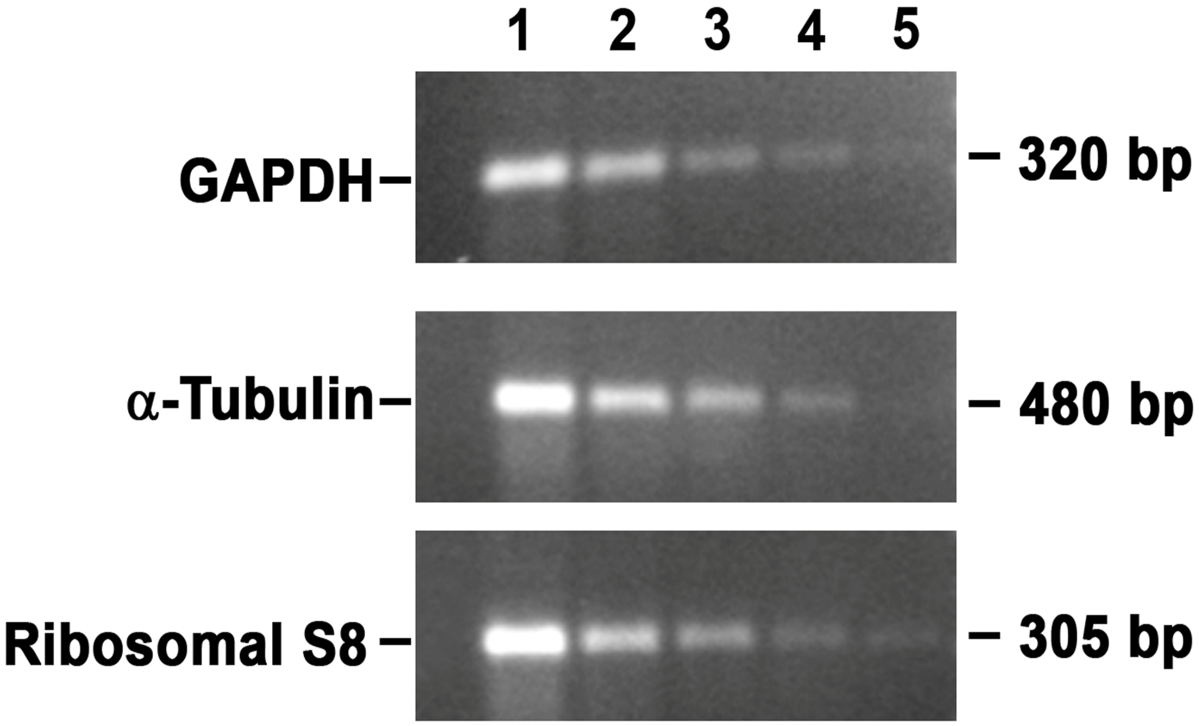

Figure S8

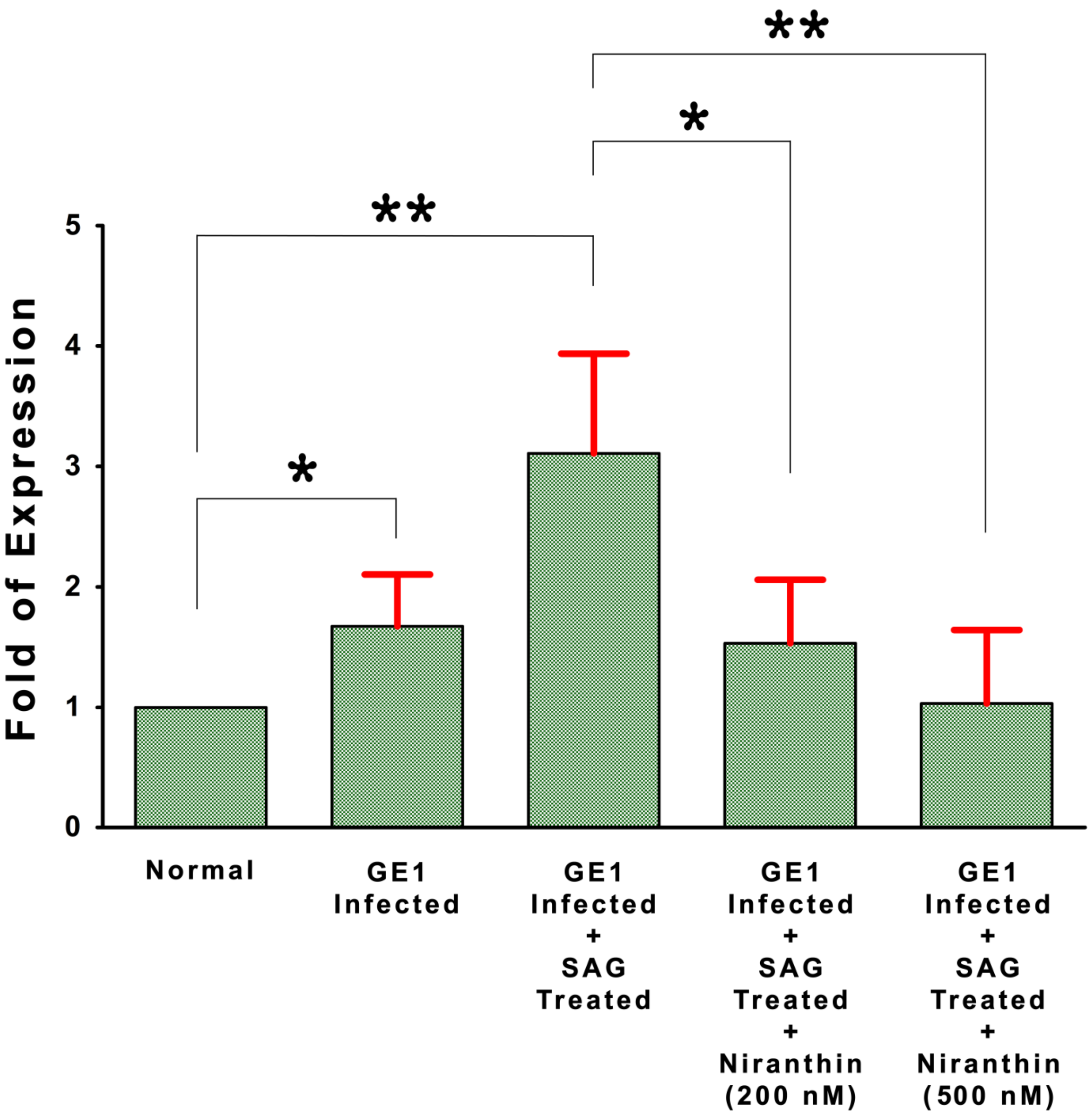
**
*
**
*
5
4
3
2
1
0
Fold of Expression
Normal
GE1 Infected
GE1 Infected + SAG Treated
GE1 Infected + SAG Treated + Niranthin (200 nM)
GE1 Infected + SAG Treated + Niranthin (500 nM)

Figure S9

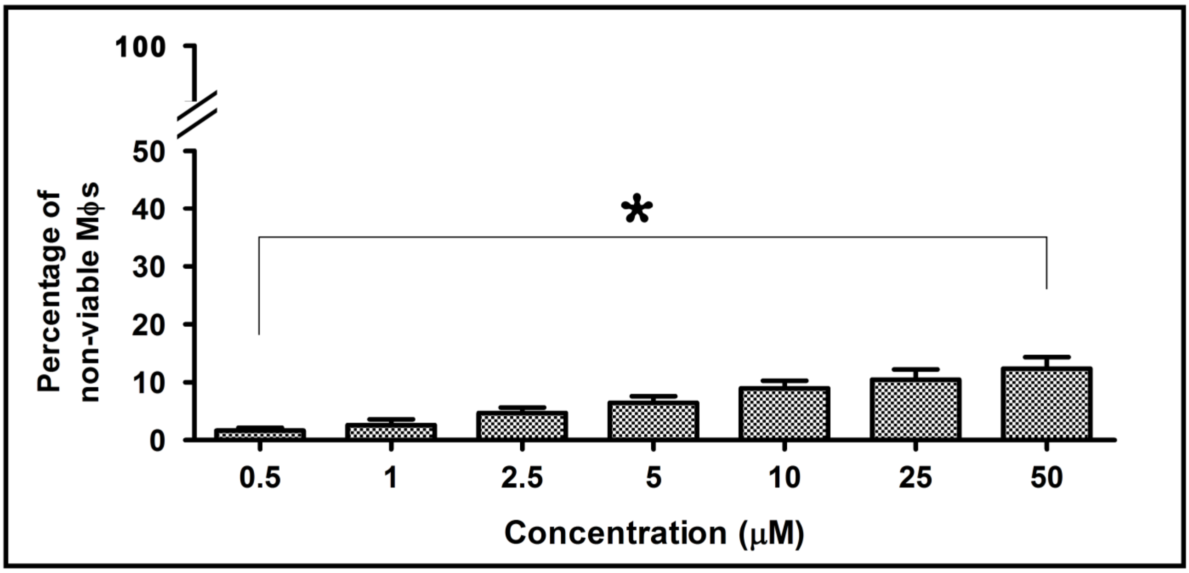
100
50
40
30
20
10
0
*
Percentage of
non-viable Mφs
0.5
1
2.5
5
10
25
50
Concentration (μM)
